# Long-term use of rozanolixizumab in generalised myasthenia gravis: final pooled analysis of the phase III MycarinG study and two open-label extensions

**DOI:** 10.1177/17562864261458532

**Published:** 2026-06-29

**Authors:** Vera Bril, Artur Drużdż, Julian Grosskreutz, Ali A. Habib, Renato Mantegazza, Sabrina Sacconi, Kimiaki Utsugisawa, Tuan Vu, Marion Boehnlein, Fiona Grimson, Niamh Houston, Virginie Kerbusch, Irene Pulido-Valdeolivas, Thaïs Tarancón, John Vissing

**Affiliations:** Ellen and Martin Prosserman Centre for Neuromuscular Diseases, Toronto General Hospital, University of Toronto, Toronto, ON M5G2C4, Canada; Department of Neurology, Municipal Hospital, Poznan´, Poland; Precision Neurology of Neuromuscular Diseases, Department of Neurology, University of Lübeck, Lübeck, Germany; MDA ALS & Neuromuscular Center, Department of Neurology, University of California, Irvine, Orange, CA, USA; Emeritus and Past Director, Department of Neuroimmunology and Neuromuscular Diseases, Fondazione IRCCS, Istituto Nazionale Neurologico Carlo Besta, Milan, Italy; Université Côte d’Azur, Peripheral Nervous System and Muscle Department, Pasteur 2 Hospital, Centre Hospitalier Universitaire de Nice, Nice, France; Department of Neurology, Hanamaki General Hospital, Hanamaki, Japan; Department of Neurology, University of South Florida Morsani College of Medicine, Tampa, FL, USA; UCB, Monheim, Germany; UCB, Slough, UK; UCB, Slough, UK; PharmAspire BV, Wijchen, The Netherlands; UCB, Madrid, Spain; UCB, Madrid, Spain; Copenhagen Neuromuscular Center, Department of Neurology, Rigshospitalet, University of Copenhagen, Copenhagen, Denmark

**Keywords:** acetylcholine receptor, cyclical treatment, FcRn, generalised myasthenia gravis, monoclonal antibody, muscle-specific tyrosine kinase, myasthenia gravis, rozanolixizumab

## Abstract

**Background:**

Myasthenia gravis (MG) is a rare autoimmune disease characterised by fluctuating and fatigable muscle weakness. In the randomised, double-blind phase III MycarinG study, one 6-week rozanolixizumab cycle significantly improved MG-specific outcomes versus placebo and was generally well tolerated in patients with generalised MG (gMG).

**Objectives:**

To assess the efficacy and safety of cyclic rozanolixizumab treatment.

**Design:**

A pooled analysis of the MycarinG, MG0004 and MG0007 studies.

**Methods:**

Following MycarinG, eligible patients could enrol in the open-label extension studies MG0004 or MG0007 to receive rozanolixizumab 7 or 10 mg/kg. In MG0004, patients received chronic weekly treatment for ⩽52 weeks. In MG0007, after an initial 6-week treatment cycle, subsequent cycles were based on symptom worsening (investigator’s discretion). Final efficacy data were pooled across MycarinG, MG0004 (first 6 weeks) and MG0007 for patients receiving ⩾2 symptom-driven cycles. Efficacy endpoints included change from baseline (CFB) in MG Activities of Daily Living (MG-ADL), MG Composite (MGC) and Quantitative MG (QMG) scores. Safety outcomes were assessed in patients who received ⩾1 cycle with a ⩽8-week follow-up period across MycarinG and MG0007.

**Results:**

Overall, 188 patients received ⩾1 cycle and 129 received ⩾2 symptom-driven cycles. Across Cycles 1–13, mean (standard deviation) CFB to Day 43 in MG-ADL score ranged from −3.2 (3.3 (*n* = 113; Cycle 3)) to −6.0 (3.9 (*n* = 24; Cycle 12)). Consistent improvements in MGC and QMG scores were also observed across repeated cycles. Treatment-emergent adverse events (TEAEs) were experienced by 175/188 (93.1%) patients; most mild or moderate. Incidence remained stable with repeated cyclic treatment among patients who remained in the study at each cycle. The most common TEAE was headache (*n* = 94/188 (50.0%)).

**Conclusion:**

Repeated rozanolixizumab treatment cycles demonstrated consistent, clinically meaningful improvements in MG-specific outcomes as early as 1 week after the first infusion. Rozanolixizumab was generally well tolerated with an acceptable safety profile, supporting its long-term use as a treatment option for adults with gMG.

**Trial registration:**

ClinicalTrials.gov: NCT03971422; NCT04124965; NCT04650854.

## Plain language summary

Long-term treatment with cycles of rozanolixizumab improved symptoms in patients with generalised myasthenia gravis in a combined analysis of final data from the MycarinG study and its two extension studies

Generalised myasthenia gravis (gMG) is an autoimmune disease that damages the connections between nerves and muscles, causing muscle weakness. In the MycarinG study, treatment with rozanolixizumab once a week for 6 weeks was better at improving gMG symptoms than placebo in adults with gMG. After MycarinG, patients could enter the extension studies MG0004 and MG0007. These studies assessed the side effects of long-term rozanolixizumab treatment and measured patients’ symptoms to see whether rozanolixizumab remained effective. In MG0004, patients received rozanolixizumab once a week for up to 52 weeks. In MG0007, patients received rozanolixizumab once a week for 6 weeks, termed a treatment cycle. After the first treatment cycle, patients only received more cycles if their symptoms worsened. We looked at data from patients who received repeated rozanolixizumab treatment cycles across MycarinG, MG0004 (first 6 weeks only) and MG0007. Treatment side effects and gMG symptoms were assessed. Overall, 129 patients received two or more rozanolixizumab cycles due to worsening symptoms. We saw consistent improvements in gMG symptoms across multiple measures; improvements were maintained over repeated treatment cycles. Altogether, we assessed 188 patients for side effects; 175 (93.1%) reported a side effect, most of which were mild or moderate in severity. The most common side effect was headache. The number of reported side effects and how bad they were did not change much across treatment cycles among patients who stayed in the study at each cycle. In the first year of treatment, patients had an average of four treatment cycles. Based on this, rozanolixizumab treatment would be expected to follow a repeated pattern of 6 weeks on treatment and 6–8 weeks off in the first year. Together, these data suggest that repeated rozanolixizumab cycles can be used for long-term treatment in patients with gMG.

## Introduction

Myasthenia gravis (MG) is a rare, chronic neuromuscular autoimmune disease characterised by fluctuating muscle weakness that significantly impacts patients’ lives.^1,2^ The disease is caused by pathogenic immunoglobulin G (IgG) autoantibodies that impair neurotransmission at the postsynaptic membrane of neuromuscular junctions.^1^ The majority of patients with MG have detectable autoantibodies directed against acetylcholine receptors (AChRs).^1^ A smaller proportion (5%–8%) have autoantibodies that target muscle-specific tyrosine kinase (MuSK).^3,4^

Rozanolixizumab is a humanised, high-affinity, high-specificity IgG4 monoclonal antibody that blocks the IgG binding region of the neonatal fragment crystallisable receptor (FcRn), whilst sparing other immunoglobulin (Ig) isotypes, such as IgA, IgM and IgE.^5,6^ Two high-affinity fragment antigen-binding (Fab) arms and two intact FcRn binding sites enable rozanolixizumab to bind up to four FcRn molecules simultaneously.^7^ By inhibiting IgG salvage and recycling, IgG destruction is accelerated, thereby reducing serum IgG levels, including levels of pathogenic IgG autoantibodies, by up to 79%.^5,8,9^ In addition to its role in IgG recycling, FcRn is also responsible for maintaining albumin levels.^8,9^ However, as rozanolixizumab binds FcRn remotely from the albumin binding site, albumin salvage remains intact.^9^

The effects of specific FcRn antagonists vary based on factors such as antibody format, structure, ability to engage other immune effector molecules, specific epitope for each antagonist on FcRn and pH-dependency.^7,10^ The binding affinity of rozanolixizumab is in the picomolar range and >600-fold higher than that of other anti-FcRn blockers.^7^ Further, the efficient recycling of rozanolixizumab is prevented owing to the lack of a clear pH-dependency in its binding to FcRn.^5,7^ This is reflected in the non-linear pharmacokinetic profile of rozanolixizumab and is typically evidence of target-mediated drug deposition.^7^

Rozanolixizumab is approved in the United States for the treatment of adult patients with anti-AChR antibody positive (Ab+) or anti-MuSK Ab+ generalised MG (gMG), in Europe as an add-on to standard therapy for the same patient population, and in Japan for the treatment of patients with gMG who respond inadequately to steroids or other immunosuppressants.^11–13^ Approval has also been received by a number of other regulatory bodies worldwide.^14–16^ These regulatory approvals were granted based on results from the randomised, double-blind, placebo-controlled phase III MycarinG study (MG0003; NCT03971422; EudraCT 2019-000968-18). This pivotal study demonstrated that a single 6-week cycle of once-weekly rozanolixizumab treatment in patients with anti-AChR Ab+ or anti-MuSK Ab+ gMG improved MG-specific outcomes versus placebo.^9^ Rozanolixizumab was generally well tolerated and had an acceptable safety profile.^9^

Following MycarinG, two phase III open-label extension (OLE) studies were initiated.^17,18^ Patients could enrol in MG0004 (NCT04124965; EudraCT 2019-000969-21) to receive chronic weekly rozanolixizumab treatment, or in MG0007 (NCT04650854; EudraCT 2020-003230-20) to receive repeated 6-week symptom-driven rozanolixizumab treatment cycles.^17,18^ An interim analysis of data pooled across MycarinG, MG0004 and MG0007 (data cut-off: 08 July 2022) has been reported previously.^18,19^ We report final pooled data on the long-term safety, tolerability and efficacy of cyclic rozanolixizumab treatment across MycarinG, MG0004 and MG0007 in patients with gMG.

## Methods

### Study design

Complete study designs for the MycarinG, MG0004 and MG0007 studies have previously been published.^9,17–19^ In brief, MycarinG was a randomised, double-blind, placebo-controlled phase III study in adults with anti-AChR Ab+ or anti-MuSK Ab+ gMG. Patients had MG Foundation of America Disease Class II–IVa gMG, an MG Activities of Daily Living (MG-ADL) score ⩾3 (for non-ocular symptoms) and a Quantitative MG (QMG) score ⩾11.^9^ Patients were randomised 1:1:1 to receive subcutaneous infusions of rozanolixizumab 7 mg/kg, rozanolixizumab 10 mg/kg or placebo once weekly for 6 weeks, followed by an 8-week observation period.^9^

**Figure 1.**
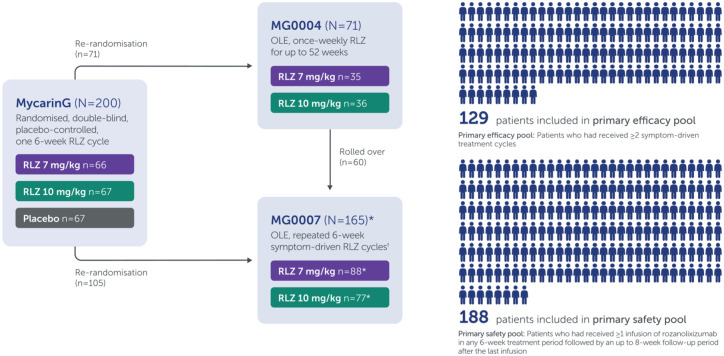
Patient disposition of primary efficacy and safety pools. *N numbers assigned in Cycle 1. ^†^After the initial cycle, dose modifications from 10 to 7 mg/kg and vice versa were permitted at the beginning of each treatment cycle, provided the benefit–risk ratio remained favourable for the patient. OLE, open-label extension; RLZ, rozanolixizumab.

Patients who completed the observation period of MycarinG or required (but chose not to receive) rescue therapy during the observation period were eligible to enrol into MG0004 and then MG0007, or into MG0007 directly (Figure 1). In MG0004, patients from MycarinG were re-randomised 1:1 to receive once-weekly rozanolixizumab 7 or 10 mg/kg for up to 52 weeks.^17^ In contrast, MG0007 comprised an initial 6-week cycle of weekly rozanolixizumab 7 or 10 mg/kg, followed by repeated symptom-driven cycles.^18^ A symptom-driven cycle was defined as a rozanolixizumab treatment cycle received following symptom worsening. Symptom worsening was at the investigator’s discretion, with examples of a ⩾2-point increase in MG-ADL score or a ⩾3-point increase in QMG score provided in the protocol.^18^ Interactive response technology (IRT) was used to assign eligible patients to a treatment dose in MG0004 and MG0007 based on a predetermined schedule produced by the IRT vendor. Only patients entering MG0007 from MycarinG were re-randomised. Patients entering MG0007 from MG0004 moved directly into the observation period of MG0007, receiving a symptom-driven cycle if their symptoms worsened at the same dose level they received in MG0004. After the initial cycle in MG0007, dose adjustments between rozanolixizumab 7 and 10 mg/kg and vice versa were permitted in between cycles.

### Study population

Data were pooled across MycarinG, MG0004 and MG0007; the pooling strategy has previously been reported^18,19^ and is summarised in Supplemental Table 1. The primary efficacy pool included patients who received ⩾2 symptom-driven rozanolixizumab cycles across MycarinG, MG0004 (first 6 weeks) and MG0007. The primary safety pool included rozanolixizumab-treated patients from MycarinG and MG0007 who received ⩾1 infusion of rozanolixizumab in any 6-week treatment period (classed as ⩾1 treatment cycle) with a subsequent follow-up period of up to 8 weeks after their last infusion. The immunogenicity pool included patients who only received cyclic rozanolixizumab treatment, with no chronic weekly treatment interruption (i.e. treatment in MG0004).

Data are reported for combined 7 and 10 mg/kg dose groups to allow for dose changes due to re-randomisation or dose switching during MG0004 and MG0007. Data reported by dose group are presented in the Supplemental Material.

### Outcomes

Key efficacy outcomes included mean change from baseline to Day 43 of each cycle in MG-ADL (primary endpoint in MycarinG), MG composite (MGC), QMG and MG Symptoms Patient-Reported Outcome (PRO) Muscle Weakness Fatigability, Physical Fatigue and Bulbar Muscle Weakness scores; the proportion of responders at Day 43 of each cycle for MG-ADL (⩾2-point improvement), QMG (⩾3-point improvement), MGC (⩾3-point improvement) and MG Symptoms PRO (Muscle Weakness Fatigability (⩾16.67-point improvement), Physical Fatigue and Bulbar Muscle Weakness (⩾20.00-point improvement)) measures; the proportion of patients achieving minimal symptom expression (MSE; MG-ADL score of 0 or 1) at any time during each cycle; and median time to MG-ADL response in each cycle. Median treatment-free interval lengths, defined as the median time from the last infusion of the previous cycle to the first infusion of the subsequent cycle, were also assessed. Efficacy data are reported for up to 13 cycles.

Pharmacodynamic outcomes included change from baseline in total IgG serum concentration. The immunogenic potential of rozanolixizumab and risk of immunogenicity-related clinical consequences were assessed via monitoring of anti-drug antibody (ADA) formation. Safety outcomes included exposure to rozanolixizumab based on the number of infusions, number of cycles and annualised number of cycles received; and incidence of treatment-emergent adverse events (TEAEs), including serious TEAEs and TEAEs leading to discontinuation of the study. TEAEs were defined as events occurring during the 6-week treatment period or ⩽8 weeks after the last dose. Events occurring after the 8-week safety follow-up period but prior to the initiation of a new rozanolixizumab treatment cycle, a period termed the intermittent period, were considered non-TEAEs.

Unless otherwise specified, efficacy and pharmacodynamic outcomes were assessed in the primary efficacy pool. Safety outcomes, including exposure, were assessed in the primary safety pool and immunogenicity was assessed in the immunogenicity pool.

### Statistical analysis

All eligible patients from the MycarinG study were invited to participate in MG0004 and MG0007, and no formal sample size calculation was performed. Data were analysed descriptively, using frequency analyses of dichotomous and categorical variables displaying the number of observations and associated percentages. For continuous variables, the number of observations was generated, as well as mean, standard deviation (SD), median, minimum and maximum values. Analysis was performed using SAS^®^ version 9.4 or later (SAS Institute Inc., Cary, NC, USA). The study protocols and statistical analysis plans were published on ClinicalTrials.gov (NCT03971422 (registered 29 May 2019); NCT04124965 (registered 11 October 2019); and NCT04650854 (registered 05 November 2020)).

Efficacy analyses for repeated treatment cycles were based on observed data in individual cycles with no imputation of missing values. Patients who received rescue therapy or were withdrawn from treatment due to TEAEs were censored at the time of rescue or onset of the TEAE. For data presented by dose, allocation of patients to treatment groups was according to the highest dose received during each cycle.

For safety analyses, adverse events were classified using the Medical Dictionary for Regulatory Activities (MedDRA^®^, Herndon, VA, USA) version 24.0.

## Results

### Patients and exposure

The MycarinG, MG0004 and MG0007 studies were conducted between 03 June 2019 and 25 January 2024. In total, 188 patients received ⩾1 treatment cycle (primary safety pool; Figure 1). Patient demographics and baseline characteristics (Supplemental Table 2) were similar to those previously reported.^19^ The mean (SD) age was 52.5 (16.3) years, 59.0% of patients were female, and baseline disease characteristics indicated a broad population of patients with gMG. Most patients (79.8%) had no history of intravenous immunoglobulin or plasma exchange use. Patients received a total of 6072 rozanolixizumab infusions (median 24.0 per patient (range 1–104)) and 1094 treatment cycles (median 4.5 per patient (range 1–18)). Mean (SD) total time in the studies was 602.3 (331.0) days (median 728.5 days (range 50–1138)), equating to 310.25 patient-years.

Overall, 129 patients received ⩾2 symptom-driven cycles (primary efficacy pool). Patient demographics and baseline characteristics (Table 1) were similar to those reported in the interim analysis,^18^ and comparable to the primary safety pool (Supplemental Table 2).

**Table 1.** Patient demographics and baseline characteristics.

Baseline characteristic	RLZ 7 mg/kg (*N* = 70)	RLZ 10 mg/kg (*N* = 59)	RLZ total (*N* = 129)
Age, years, mean (SD)	52.2 (14.3)	49.3 (18.3)	50.9 (16.3)
Sex, female, *n* (%)	41 (58.6)	36 (61.0)	77 (59.7)
Body weight, *n* (%)			
<50 kg	8 (11.4)	2 (3.4)	10 (7.8)
50 to <70 kg	16 (22.9)	24 (40.7)	40 (31.0)
70 to <100 kg	33 (47.1)	20 (33.9)	53 (41.1)
⩾100 kg	13 (18.6)	13 (22.0)	26 (20.2)
BMI, kg/m^2^, mean (SD)	27.3 (6.7)	27.6 (6.2)	27.4 (6.5)
Geographic region, *n* (%)			
North America	20 (28.6)	10 (16.9)	30 (23.3)
Europe	40 (57.1)	44 (74.6)	84 (65.1)
Asia (excl. Japan)	2 (2.9)	1 (1.7)	3 (2.3)
Japan	8 (11.4)	4 (6.8)	12 (9.3)
Race, *n* (%)*			
Asian	10 (14.3)	5 (8.5)	15 (11.6)
Black	0	1 (1.7)	1 (0.8)
Native Hawaiian or other Pacific Islander	0	0	0
White	42 (60.0)	43 (72.9)	85 (65.9)
Missing	18 (25.7)	10 (16.9)	28 (21.7)
Age at initial gMG diagnosis, years, mean (SD)	44.7 (15.8)	41.2 (19.9)	43.1 (17.8)
Duration of disease, years, mean (SD)	7.8 (8.3)	8.4 (8.8)	8.1 (8.5)
MGFA disease class, *n* (%)			
II	33 (47.1)	19 (32.2)	52 (40.3)
III	34 (48.6)	38 (64.4)	72 (55.8)
IV	3 (4.3)	2 (3.4)	5 (3.9)
MG crisis, *n* (%)	18 (25.7)	13 (22.0)	31 (24.0)
Thymectomy, *n* (%)	33 (47.1)	22 (37.3)	55 (42.6)
Anti-AChR Ab+, *n* (%)	62 (88.6)	55 (93.2)	117 (90.7)
Anti-MuSK Ab+, *n* (%)	9 (12.9)	3 (5.1)	12 (9.3)
MG-ADL score, mean (SD)	9.0 (3.8)	8.4 (2.9)	8.7 (3.4)
QMG score, mean (SD)	16.0 (3.8)	16.1 (3.7)	16.0 (3.8)
Total IgG, g/L, mean (SD)	10.3 (2.7)	9.9 (2.7)	10.1 (2.7)
No prior IVIg or PLEX, *n* (%)	54 (77.1)	42 (71.2)	96 (74.4)
Baseline gMG medication, *n* (%)			
CS for systemic use	42 (60.0)	38 (64.4)	80 (62.0)
Immunosuppressants	35 (50.0)	31 (52.5)	66 (51.2)
Parasympathomimetics	60 (85.7)	53 (89.8)	113 (87.6)

Primary efficacy pool.

* Data on race were not permitted to be collected in France and Canada.

Ab+, antibody positive; AChR, acetylcholine receptor; BMI, body mass index; CS, corticosteroid; gMG, generalised myasthenia gravis; IgG, immunoglobulin G; IVIg, intravenous immunoglobulin; MG, myasthenia gravis; MG-ADL, Myasthenia Gravis Activities of Daily Living; MGFA, Myasthenia Gravis Foundation of America; MuSK, muscle-specific tyrosine kinase; PLEX, plasma exchange; QMG, Quantitative Myasthenia Gravis; RLZ, rozanolixizumab; SD, standard deviation.

### Efficacy

**Figure 2.**
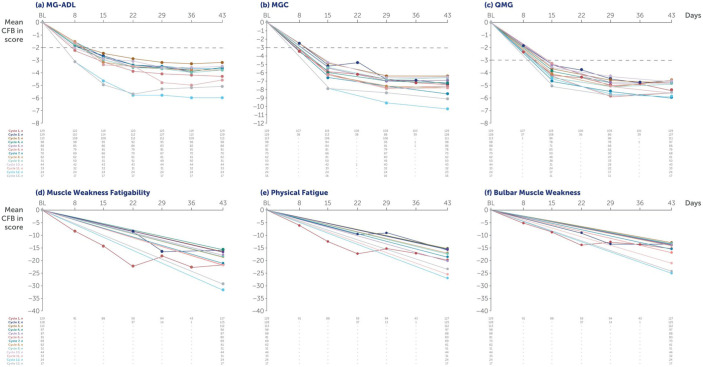
Mean change from baseline to Day 43 in (a) MG-ADL, (b) MGC, (c) QMG, (d) MG symptoms PRO Muscle Weakness Fatigability, (e) MG Symptoms PRO Physical Fatigue and (f) MG Symptoms PRO Bulbar Muscle Weakness scores. Primary efficacy pool. Efficacy data collected at or after the time point of rescue use were excluded from the analysis with no imputation of missing data for the respective cycle. The reference values for meaningful change were a 2-point improvement for MG-ADL and a 3-point improvement for MGC and QMG. The reference values for meaningful within-patient change for the MG Symptoms PRO scales were a 16.67-point improvement for Muscle Weakness Fatigability and a 20.00-point improvement for Physical Fatigue and Bulbar Muscle Weakness. BL, baseline; CFB, change from baseline; MG Symptoms PRO, Myasthenia Gravis Symptoms Patient-Reported Outcome; MG-ADL, Myasthenia Gravis Activities of Daily Living; MGC, Myasthenia Gravis Composite; QMG, Quantitative Myasthenia Gravis.

Compared with Cycle 1, improvements from baseline in MG-ADL score showed a robust, consistent profile over each subsequent cycle (Figure 2). A rapid decrease in MG-ADL score was observed at Day 8, reaching a maximum between Day 29 and Day 43 that was clinically meaningful. Across Cycles 1 to 13, mean (SD) change from baseline to Day 43 ranged from −3.2 (3.3; *n* = 113) in Cycle 3 to −6.0 (3.9; *n* = 24) in Cycle 12. Consistent improvements from baseline in MGC, QMG and MG Symptoms PRO scale scores were observed across repeated treatment cycles, with improvements observed as early as 1 week after the first infusion in Cycle 1 (Figure 2). Similar reductions in MG-specific outcomes were observed for the 7 and 10 mg/kg dose groups (Supplemental Figure 1).

Improvements in MG-ADL score were consistent between subgroups of patients with anti-AChR Ab+ and anti-MuSK Ab+ gMG. In patients with anti-AChR Ab+ gMG, the mean (SD) change from baseline to Day 43 across Cycles 1 to 13 ranged from −3.2 (3.2; *n* = 105) in Cycle 3 to −5.9 (3.9; *n* = 22) in Cycle 12. In patients with anti-MuSK Ab+ gMG, improvements in MG-ADL score to Day 43 ranged from −3.0 (3.6; *n* = 8) in Cycle 5 to −7.0 (3.5; *n* = 12) in Cycle 1.

**Figure 3.**
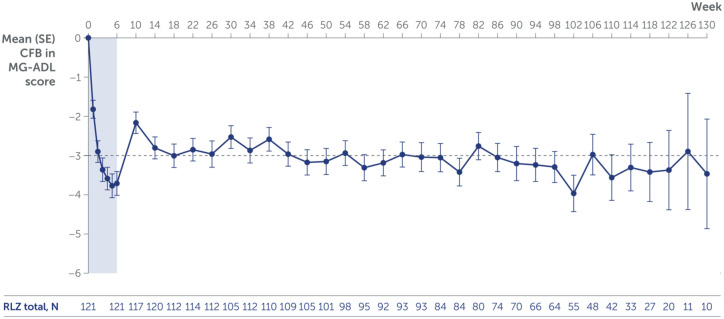
Mean change from baseline in MG-ADL score to Week 130. Pool E3; consisting of patients who received ⩾2 consecutive symptom-driven treatment cycles. Data at Weeks 0–6 represent observed MG-ADL scores in the Cycle 1 treatment period. After this, patients followed their own cadence of rozanolixizumab treatment cycles, with average monthly (28-day) MG-ADL scores calculated for each patient. The group-level average was calculated using data from all patients with at least one MG-ADL measurement at that time period. If patients had multiple MG-ADL measurements during the period, their average MG-ADL score over the measurements was used. CFB, change from baseline; MG-ADL, Myasthenia Gravis Activities of Daily Living; RLZ, rozanolixizumab; SE, standard error.

At the population level, as individual patients cycled through treatment at their own frequency, a mean improvement in MG-ADL score of approximately 3.0 points from baseline was maintained over 130 weeks of repeated rozanolixizumab treatment cycles (Pool E3; Figure 3). For QMG score, a mean improvement of approximately 4.0 points from baseline was maintained.

**Figure 4.**
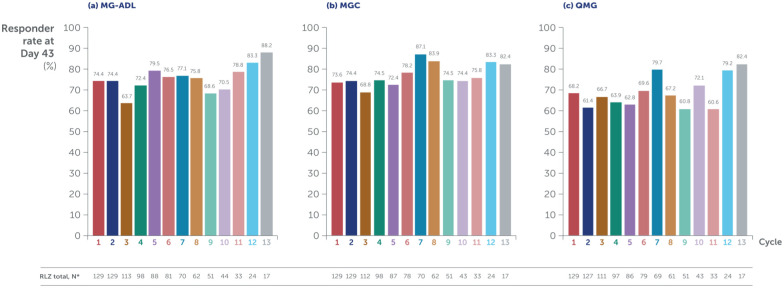
Responder rates at Day 43 during each cycle for (a) MG-ADL, (b) MGC and (c) QMG scores. Primary efficacy pool. MG-ADL, MGC and QMG response are defined as a ⩾2-point, ⩾3-point and ⩾3-point improvement in score from baseline, respectively, without rescue therapy. *N represents the number of patients who had completed an MG-ADL, MGC or QMG assessment at Day 43 in each treatment cycle. MG-ADL, Myasthenia Gravis Activities of Daily Living; MGC, Myasthenia Gravis Composite; QMG, Quantitative Myasthenia Gravis; RLZ, rozanolixizumab.

High responder rates were observed at Day 43 across MG-ADL, MGC and QMG assessments in Cycle 1, with consistent responses recorded following repeated cyclic treatment (Figure 4 and Supplemental Table 3). Based on a Kaplan–Meier estimate, the median time to MG-ADL response was ~2 weeks up to Cycle 11; variability increased from Cycle 12 onwards. Data for MG Symptoms PRO scale responders are presented in Supplemental Figure 2 and Supplemental Table 3. Achievement of MSE tended to increase across cycles and ranged from a minimum of 26.5% (*n* = 30/113) in Cycle 3 to a maximum of 47.1% (*n* = 8/17) in Cycle 13 (Supplemental Table 4).

In MycarinG, patients were required to have a stable corticosteroid dose 4 weeks prior to baseline and for the duration of the study. In MG0007, investigators could reduce, increase, start or stop corticosteroids at their own discretion, with no guidance on tapering regimens provided in the protocol. Of 121 patients who had received ⩾2 consecutive symptom-driven treatment cycles across MycarinG and MG0007 (Pool E3), 66.1% (*n* = 80/121) had no change in corticosteroid dose at their last available follow-up (mean dose 7.5 mg/day (range 0.0–80.0 mg/day)) and 13.2% (*n* = 16/121) had increased their corticosteroid dose, which included 5.8% (*n* = 7/121) who started corticosteroids. A total of 67 patients were receiving corticosteroids at baseline, of whom 37.3% (*n* = 25/67) had decreased their corticosteroid dose, including 17.9% (*n* = 12/67) who discontinued corticosteroids. Of 53 patients receiving a corticosteroid dose ⩾7.5 mg/day (Cushing threshold)^20^ at baseline, 41.5% (*n* = 22/53) decreased their corticosteroid dose, including 18.9% (*n* = 10/53) who discontinued corticosteroids.

Patients receiving non-steroidal immunosuppressants (NSISTs) in the 6 months prior to baseline in the MycarinG study were required to have a stable dose 2 months prior to baseline and for the duration of the study. In MG0007, investigators were encouraged to maintain stable NSIST doses throughout each 6-week cycle and 8-week follow-up period. In total, 86.0% (*n* = 104/121) of patients had no change in NSIST dose at their last available follow-up and 3.3% (*n* = 4/121) had increased the dose of ⩾1 NSIST. Among patients receiving NSISTs at baseline, 19.6% (*n* = 11/56) had decreased the dose of ⩾1 NSIST, including one patient who increased their dose of mycophenolate mofetil but decreased their dose of azathioprine.

### Treatment patterns

The number of cycles, number of infusions and time between cycles (treatment-free intervals) varied between patients. For the 188 patients in the primary safety pool, the mean (SD) annualised number of cycles initiated was 2.9 (1.8) per year and of infusions received was 16.0 (10.6) per year. Of these 188 patients, 130 (69.1%) had >1 year of participation, and they initiated a mean (SD) of 4.1 (1.7) cycles (median 4.0 (range 1–7)) and received 22.0 (8.9) infusions (median 23.0 (range 5–39)) in the first year. At the population level, based on the observed mean of approximately four cycles per year, an expected repeated treatment pattern in the first year of treatment would be a 6-week treatment period followed by a 6–8-week treatment-free interval that could be adjusted according to patient needs.

**Figure 5.**
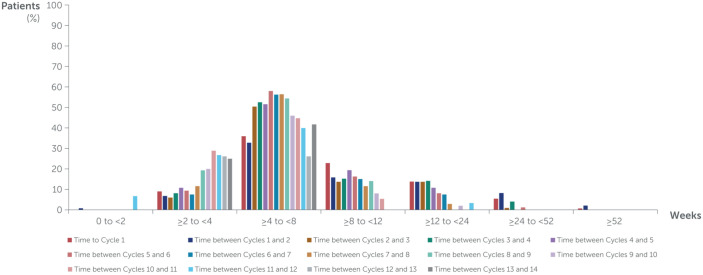
Frequency of treatment-free intervals. Pool E2; consisting of patients who received rozanolixizumab treatment and had initiated or were awaiting a symptom-driven treatment cycle. Patients without a symptom-driven cycle after rozanolixizumab treatment were censored at the time of dropping out or at the end of the study (MycarinG or MG0007). Number of censored patients: Time to Cycle 1, *n* = 21; time between Cycles 1 and 2, *n* = 29; time between Cycles 2 and 3, *n* = 18; time between Cycles 3 and 4, *n* = 6; time between Cycles 4 and 5, *n* = 7; time between Cycles 5 and 6, *n* = 6; time between Cycles 6 and 7, *n* = 11; time between Cycles 7 and 8, *n* = 12; time between Cycles 8 and 9, *n* = 7; time between Cycles 9 and 10, *n* = 12; time between Cycles 10 and 11, *n* = 8; time between Cycles 11 and 12, *n* = 7; time between Cycles 12 and 13, *n* = 11; time between Cycles 13 and 14, *n* = 4.

For the majority of cycles up to Cycle 13, the most frequently occurring treatment-free intervals were 4 to <8 weeks (Pool E2; Figure 5). Based on a Kaplan–Meier estimate, the median treatment-free interval was 9 weeks (63 days) for time to first symptom-driven cycle (Pool E2).

### Pharmacodynamics

**Figure 6.**
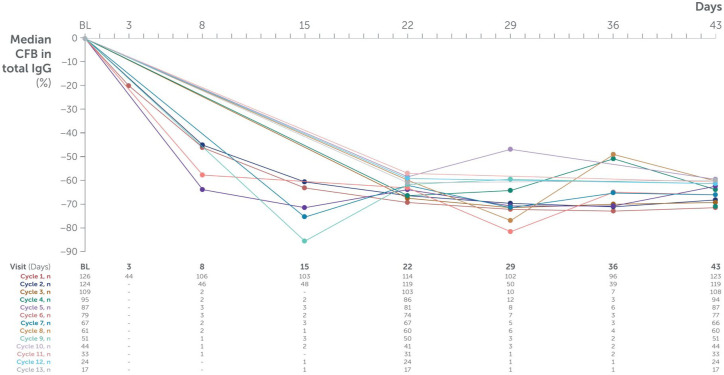
Median percentage change from baseline in total IgG serum concentration. Primary efficacy pool. BL, baseline; CFB, change from baseline; IgG, immunoglobulin.

A rapid and sustained decrease from baseline in total IgG serum concentration was observed across all cycles, with reductions seen as early as Day 3 (Cycle 1) and no evidence of loss of response in later treatment cycles (Figure 6). Across Cycles 1 to 13, median percentage change from baseline to Day 43 ranged from −59.29 (*n* = 44) in Cycle 10 to −71.45 (*n* = 123) in Cycle 1. In patients with anti-AChR Ab+ or anti-MuSK Ab+ gMG, levels of anti-AChR and anti-MuSK antibodies decreased in line with the reduction from baseline observed in total IgG levels (data not shown).

### Immunogenicity

A total of 168 patients were included in the immunogenicity pool, 156 (92.9%) of whom were included in the evaluation of ADAs. After Cycle 1, 26.9% (*n* = 42/156) of patients had developed ADAs; this increased to 61.2% (*n* = 30/49) after seven cycles. Incidence of TEAEs was generally consistent between ADA-positive (94.0% (*n* = 79/84)) and ADA-negative (86.1% (*n* = 62/72)) patients. Based on MG-ADL scores, there was no apparent impact of immunogenicity on efficacy (data not shown).

### Safety

Overall, 175 (93.1% (*n* = 188)) patients experienced a TEAE during 223.8 total patient-years at risk (Table 2). In general, incidence of TEAEs remained stable with repeated cyclic treatment among patients who remained in the study at each cycle and was higher in the 10 mg/kg group than in the 7 mg/kg group (Supplemental Table 5). Exceptions included the incidences of decreased blood IgG and coronavirus disease 2019 (COVID-19), which were increased compared with Cycle 1 in the majority of cycles, consistent with the known mechanism of action of rozanolixizumab and the conduct of the study coinciding with the COVID-19 pandemic. Headache, diarrhoea, COVID-19 and pyrexia were the most commonly reported TEAEs, occurring in ⩾20% of patients overall (Table 3).

**Table 2.** Overview of TEAEs.

TEAE type	Event rate (100 PYAR)	Cycle, *n* (%)													
		All *N* = 188*	1 *N* = 188	2 *N* = 145	3 *N* = 117	4 *N* = 102	5 *N* = 94	6 *N* = 86	7 *N* = 78	8 *N* = 71	9 *N* = 59	10 *N* = 50	11 *N* = 37	12 *N* = 26	13 *N* = 18
Any TEAEs	1023.9	175 (93.1)	149 (79.3)	104 (71.7)	76 (65.0)	65 (63.7)	70 (74.5)	63 (73.3)	52 (66.7)	42 (59.2)	34 (57.6)	31 (62.0)	23 (62.2)	11 (42.3)	11 (61.1)
Serious TEAEs^a^	42.0	55 (29.3)	20 (10.6)	9 (6.2)	5 (4.3)	6 (5.9)	8 (8.5)	2 (2.3)	5 (6.4)	5 (7.0)	1 (1.7)	3 (6.0)	1 (2.7)	1 (3.8)	2 (11.1)
MG worsening	-	18 (9.6)	5 (2.7)	2 (1.4)	2 (1.7)	1 (1.0)	3 (3.2)	-	1 (1.3)	1 (1.4)	1 (1.7)	1 (2.0)	1 (2.7)	1 (3.8)	2 (11.1)
MG crisis	-	4 (2.1)	1 (0.5)	2 (1.4)	1 (0.9)	-	-	-	-	-	-	-	-	-	-
Permanent discontinuation of study due to TEAEs	17.0	33 (17.6)	13 (6.9)	8 (5.5)	3 (2.6)	3 (2.9)	1 (1.1)	-	1 (1.3)	1 (1.4)	-	2 (4.0)	1 (2.7)	-	-
Treatment-related TEAEs	416.5	120 (63.8)	94 (50.0)	51 (35.2)	26 (22.2)	32 (31.4)	30 (31.9)	32 (37.2)	24 (30.8)	21 (29.6)	18 (30.5)	14 (28.0)	12 (32.4)	4 (15.4)	4 (22.2)
Severe TEAEs^a^	51.8	62 (33.0)	23 (12.2)	9 (6.2)	6 (5.1)	9 (8.8)	9 (9.6)	6 (7.0)	3 (3.8)	5 (7.0)	3 (5.1)	5 (10.0)	1 (2.7)	1 (3.8)	2 (11.1)
MG worsening	-	16 (8.5)	5 (2.7)	1 (0.7)	2 (1.7)	1 (1.0)	3 (3.2)	-	1 (1.3)	1 (1.4)	1 (1.7)	1 (2.0)	1 (2.7)	1 (3.8)	2 (11.1)
Headache	-	8 (4.3)	7 (3.7)	-	-	-	-	1 (1.2)	-	-	1 (1.7)	1 (2.0)	-	-	-
MG crisis	-	4 (2.1)	1 (0.5)	2 (1.4)	1 (0.9)	-	-	-	-	-	-	-	-	-	-
Decreased blood IgG	-	4 (2.1)	-	-	1 (0.9)	3 (2.9)	1 (1.1)	-	-	-	-	-	-	-	-
TEAEs leading to death	1.8	4 (2.1)	-	2 (1.4)	1 (0.9)	-	-	-	1 (1.3)	-	-	-	-	-	-

Primary safety pool. *n* is the number of patients reporting ⩾1 TEAE within the category and cycle.

* Sum of cycles = 1094. Data past Cycle 13 may be included.

a The individual TEAEs presented underneath ‘Serious TEAEs’ and ‘Severe TEAEs’ include those reported for ⩾2% of patients in the ‘All’ column.

IgG, immunoglobulin G; MG, myasthenia gravis; PYAR, patient-years at risk; TEAE, treatment-emergent adverse event.

**Table 3.** Most common TEAEs.

TEAE type	Event rate (100 PYAR)	Cycle, *n* (%)													
		All *N* = 188^*^	1 *N* = 188	2 *N* = 145	3 *N* = 117	4 *N* = 102	5 *N* = 94	6 *N* = 86	7 *N* = 78	8 *N* = 71	9 *N* = 59	10 *N* = 50	11 *N* = 37	12 *N* = 26	13 *N* = 18
Any TEAEs^a^	1023.9	175 (93.1)	149 (79.3)	104 (71.7)	76 (65.0)	65 (63.7)	70 (74.5)	63 (73.3)	52 (66.7)	42 (59.2)	34 (57.6)	31 (62.0)	23 (62.2)	11 (42.3)	11 (61.1)
Headache	189.0	94 (50.0)	69 (36.7)	34 (23.4)	21 (17.9)	18 (17.6)	14 (14.9)	15 (17.4)	16 (20.5)	10 (14.1)	13 (22.0)	7 (14.0)	5 (13.5)	-	1 (5.6)
Diarrhoea	62.6	63 (33.5)	36 (19.1)	11 (7.6)	7 (6.0)	10 (9.8)	11 (11.7)	10 (11.6)	4 (5.1)	7 (9.9)	4 (6.8)	3 (6.0)	1 (2.7)	-	1 (5.6)
COVID-19	20.6	41 (21.8)	4 (2.1)	11 (7.6)	6 (5.1)	5 (4.9)	3 (3.2)	6 (7.0)	3 (3.8)	2 (2.8)	1 (1.7)	1 (2.0)	2 (5.4)	-	-
Pyrexia	40.2	39 (20.7)	25 (13.3)	9 (6.2)	2 (1.7)	5 (4.9)	2 (2.1)	5 (5.8)	4 (5.1)	3 (4.2)	4 (6.8)	3 (6.0)	3 (8.1)	-	2 (11.1)
Nausea	25.9	33 (17.6)	15 (8.0)	9 (6.2)	6 (5.1)	4 (3.9)	5 (5.3)	2 (2.3)	1 (1.3)	2 (2.8)	2 (3.4)	1 (2.0)	1 (2.7)	-	1 (5.6)
MG worsening	17.4	26 (13.8)	9 (4.8)	2 (1.4)	2 (1.7)	2 (2.0)	5 (5.3)	1 (1.2)	2 (2.6)	2 (2.8)	1 (1.7)	2 (4.0)	1 (2.7)	1 (3.8)	2 (11.1)
Arthralgia	13.4	23 (12.2)	10 (5.3)	4 (2.8)	3 (2.6)	6 (5.9)	1 (1.1)	2 (2.3)	-	1 (1.4)	-	1 (2.0)	-	-	-
Nasopharyngitis	16.1	23 (12.2)	7 (3.7)	-	5 (4.3)	4 (3.9)	3 (3.2)	2 (2.3)	2 (2.6)	6 (8.5)	1 (1.7)	4 (8.0)	1 (2.7)	-	-
Decreased blood IgG	17.9	21 (11.2)	4 (2.1)	8 (5.5)	5 (4.3)	7 (6.9)	5 (5.3)	4 (4.7)	2 (2.6)	2 (2.8)	-	-	1 (2.7)	-	-
Abdominal pain	10.7	19 (10.1)	5 (2.7)	5 (3.4)	2 (1.7)	3 (2.9)	-	2 (2.3)	-	1 (1.4)	2 (3.4)	-	-	1 (3.8)	-
URTI	17.0	19 (10.1)	3 (1.6)	4 (2.8)	1 (0.9)	1 (1.0)	4 (4.3)	4 (4.7)	4 (5.1)	2 (2.8)	2 (3.4)	4 (8.0)	1 (2.7)	2 (7.7)	1 (5.6)

Primary safety pool. *n* is the number of patients reporting ⩾1 TEAE within the category and cycle.

* Sum of cycles = 1094. Data past Cycle 13 may be included.

a The individual TEAEs presented underneath ‘Any TEAEs’ include those reported for ⩾10% of patients in the ‘All’ column.

COVID-19, coronavirus disease 2019; IgG, immunoglobulin G; MG, myasthenia gravis; PYAR, patient-years at risk; TEAE, treatment-emergent adverse event; URTI, upper respiratory tract infection.

In total, 62 (33.0%) patients experienced TEAEs of severe intensity, and serious TEAEs were experienced by 55 (29.3%) patients (Table 2). Across all cycles, serious TEAEs occurring in >1 patient were MG worsening (18 (9.6%)), MG crisis (4 (2.1%)), COVID-19 (3 (1.6%)), nephrolithiasis (2 (1.1%)) and pneumonia (2 (1.1%)). In general, incidence of severe and serious TEAEs remained stable with repeated cyclic treatment among patients who remained in the study at each cycle and was higher in the 10 mg/kg group than in the 7 mg/kg group.

Non-TEAEs, occurring after the 8-week safety follow-up period for each cycle but prior to the first infusion of the next rozanolixizumab cycle, occurred in 76 (40.4%) patients. Serious non-TEAEs were reported in 15 (8.0%) patients (25 events) and treatment-related non-TEAEs in five (2.7%) patients (five events).

Permanent discontinuation from the study due to TEAEs was reported in 33 (17.6%) patients, with the majority of events occurring in Cycles 1 and 2 (Table 2). Incidence remained low throughout repeated cycles of treatment (<7% of patients per cycle) among patients who remained in the study at each cycle. TEAEs leading to study discontinuation reported in >1 patient were MG worsening (5 (2.7%)), MG crisis (2 (1.1%)) and positive interferon gamma release assay (2 (1.1%)).

A total of 97 (51.6%) patients experienced any headache (including migraine and migraine with aura), and headaches were predominantly mild or moderate in intensity (Table 4). When necessary, headaches were generally well managed with over-the-counter non-opioid analgesics. Headache was reported in 94 (50.0%) patients, migraine in six (3.2%) patients and migraine with aura in one (0.5%) patient. Compared with Cycle 1, incidence of any headache was stable with repeated treatment cycles among patients who remained in the study. Most severe headaches occurred in Cycle 1 (Table 4). Three severe headaches, one event each in Cycles 6, 9 and 10, occurred in the same patient; none of the three events resulted in study discontinuation and one was considered unrelated to treatment. One patient permanently discontinued the study due to a TEAE of headache.

**Table 4.** Incidence of headache, GI disturbance, hypersensitivity reactions, injection site reactions and infections.

TEAE type	Cycle, *n* (%)													
	All, *N* = 188*	1, *N* = 188	2, *N* = 145	3, *N* = 117	4, *N* = 102	5, *N* = 94	6, *N* = 86	7, *N* = 78	8, *N* = 71	9, *N* = 59	10, *N* = 50	11, *N* = 37	12, *N* = 26	13, *N* = 18
Any headache^a^	97 (51.6)	72 (38.3)	34 (23.4)	21 (17.9)	20 (19.6)	14 (14.9)	15 (17.4)	16 (20.5)	10 (14.1)	13 (22.0)	7 (14.0)	5 (13.5)	-	1 (5.6)
Serious	1 (0.5)	1 (0.5)	-	-	-	-	-	-	-	-	-	-	-	-
Severe	8 (4.3)	7 (3.7)	-	-	-	-	1 (1.2)	-	-	1 (1.7)	1 (2.0)	-	-	-
Any GI disturbance	82 (43.6)	51 (27.1)	21 (14.5)	14 (12.0)	14 (13.7)	14 (14.9)	13 (15.1)	10 (12.8)	9 (12.7)	8 (13.6)	4 (8.0)	2 (5.4)	1 (3.8)	2 (11.1)
Serious	2 (1.1)	2 (1.1)	-	-	-	-	-	-	-	-	-	-	-	-
Severe	4 (2.1)	3 (1.6)	-	-	-	-	-	-	1 (1.4)	-	-	-	-	-
Any hypersensitivity reaction	29 (15.4)	13 (6.9)	7 (4.8)	2 (1.7)	3 (2.9)	3 (3.2)	-	1 (1.3)	2 (2.8)	-	2 (4.0)	-	1 (3.8)	-
Serious	-	-	-	-	-	-	-	-	-	-	-	-	-	-
Severe	1 (0.5)	-	-	-	1 (1.0)	-	-	-	-	-	-	-	-	-
Any injection site reaction^b^	25 (13.3)	13 (6.9)	10 (6.9)	4 (3.4)	2 (2.0)	3 (3.2)	1 (1.2)	1 (1.3)	1 (1.4)	1 (1.7)	-	-	-	-
Any infection or infestation	109 (58.0)	44 (23.4)	27 (18.6)	28 (23.9)	21 (20.6)	28 (29.8)	22 (25.6)	19 (24.4)	21 (29.6)	13 (22.0)	14 (28.0)	7 (18.9)	3 (11.5)	2 (11.1)
Serious	12 (6.4)	3 (1.6)	2 (1.4)	1 (0.9)	2 (2.0)	-	1 (1.2)	1 (1.3)	2 (2.8)	-	-	-	-	-
Severe	11 (5.9)	2 (1.1)	2 (1.4)	1 (0.9)	2 (2.0)	-	1 (1.2)	1 (1.3)	2 (2.8)	-	-	-	-	-
Any opportunistic infection	4 (2.1)	-	-	1 (0.9)	-	-	1 (1.2)	-	1 (1.4)	1 (1.7)	-	-	-	-
Serious	1 (0.5)	-	-	-	-	-	1 (1.2)	-	-	-	-	-	-	-
Severe	1 (0.5)	-	-	-	-	-	1 (1.2)	-	-	-	-	-	-	-

Primary safety pool. *n* is the number of patients reporting ⩾1 TEAE within the category and cycle.

* Sum of cycles = 1094. Data past Cycle 13 may be included.

a Includes headache, migraine and migraine with aura.

b There were no serious or severe injection site reactions.

GI, gastrointestinal; TEAE, treatment-emergent adverse event.

Gastrointestinal (GI) disturbances were experienced by 82 (43.6%) patients (Table 4). Compared with Cycle 1, the incidence of GI disturbances was stable with repeated cyclic treatment among patients who remained in the study. The most common TEAEs meeting the criteria for GI disturbance were diarrhoea (63 (33.5%)) and nausea (33 (17.6%)). Serious GI disturbances occurred in two (1.1%) patients (Table 4; gastritis and vomiting in one patient each). Severe GI disturbances occurred in four (2.1%) patients (Table 4; vomiting in one patient and diarrhoea in three patients).

Infections were reported in 109 (58.0%) patients and were mostly mild or moderate in intensity (Table 4). The most common infections were COVID-19 (41 (21.8%)), upper respiratory tract infection (19 (10.1%)), nasopharyngitis (23 (12.2%)), urinary tract infection (13 (6.9%)) and oral herpes (10 (5.3%)). The most common serious infections were COVID-19 and pneumonia. Serious TEAEs of COVID-19 and pneumonia were experienced by three (1.6%) patients and two (1.1%) patients, respectively. COVID-19, pneumonia and peritonitis comprised the most common severe infections. Severe TEAEs of COVID-19, pneumonia and peritonitis occurred in two (1.1%) patients each. One case of aseptic meningitis, reported as drug-induced aseptic meningitis and deemed to be related to rozanolixizumab, was reported in Cycle 1. Four (2.1%) patients experienced opportunistic infections (Table 4); these were sinusitis aspergillus, blastocystis infection, ophthalmic herpes simplex and oesophageal candidiasis. A medical review of these cases did not identify a concern for an increased risk of opportunistic infections associated with rozanolixizumab treatment.

All hypersensitivity and injection site reactions were non-serious and were experienced by <7% of patients per cycle up to Cycle 13 (Table 4). One severe event of rash was reported in Cycle 4; the patient was subsequently diagnosed with subacute cutaneous lupus erythematosus. There were no severe injection site reactions and no anaphylactic reactions. No clinically meaningful changes in albumin or lipid levels were observed.

There were a total of six deaths, all of which occurred during MG0007. Five of these deaths were previously reported in the interim read-out.^19^ Overall, four patients experienced fatal TEAEs (Table 2), and one patient had a fatal non-TEAE. Two patients experienced fatal TEAEs related to COVID-19 (rozanolixizumab 10 mg/kg; one event of COVID-19 and one event of COVID-19 pneumonia). A further patient was hospitalised with endocarditis and developed severe-acute-respiratory syndrome-related coronavirus 2 infection. Two days after discharge the patient was diagnosed with bacterial pneumonia and suspected septic shock and subsequently died of cardiac failure (rozanolixizumab 10 mg/kg). One patient receiving prednisolone, mycophenolate mofetil and amiodarone was hospitalised 57 days after their last rozanolixizumab infusion with a serious TEAE of pneumonia. The patient subsequently developed acute kidney injury, acute respiratory failure, cardiac failure and acute respiratory distress syndrome and died (rozanolixizumab 7 mg/kg). A fatal myocardial infarction occurred in one patient 326 days after their last rozanolixizumab 10 mg/kg dose; the patient had discontinued the study approximately 6 months prior. The sixth death was a non-TEAE in a patient who received a single 7 mg/kg treatment cycle and was diagnosed with metastatic small cell lung cancer 804 days (26.5 months) after their last dose of rozanolixizumab, which progressed to a fatal outcome post-study. None of the deaths were considered as related to rozanolixizumab by investigators.

## Discussion

This integrated pooled analysis of the phase III MycarinG study and its OLE studies assessed the efficacy and safety of repeated 6-week treatment cycles with rozanolixizumab 7 or 10 mg/kg in patients with gMG. The clinical efficacy of repeated rozanolixizumab treatment cycles was demonstrated through consistent improvements across all efficacy variables, which were maintained across repeated cycles. Results were consistent with those reported in the interim analysis; rozanolixizumab had an acceptable safety profile and was generally well tolerated.^18,19^

Across each treatment cycle, improvements from baseline to Day 43 in MG-ADL, MGC and QMG scores were clinically meaningful (⩾2-point, ⩾3-point and ⩾3-point improvement, respectively).^21–23^ As measured by MG-ADL score, response to rozanolixizumab was rapid, and was consistently observed as early as Day 8 (the first post-baseline assessment) across Cycles 1 to 13. Improvements from baseline in MG-specific outcomes were also observed for the subgroup of patients with anti-MuSK Ab+ gMG, who often experience an unsatisfactory response to therapies commonly used in the treatment of anti-AChR Ab+ gMG.^1,4^

High rates (>60%) of MG-ADL, MGC and QMG responders were consistently observed over repeated treatment cycles, and at least 26% of patients achieved MSE in each cycle. MSE is a stringent measure of therapeutic efficacy^24^ and considered a desirable goal in the management of MG.^25,26^

Corticosteroids are commonly used to manage the symptoms of MG, but their chronic use is associated with a number of systemic side effects, including hypertension, an increased risk of infection, osteoporosis, weight gain, skin atrophy and mood disorders.^27^ The international consensus guidance for the management of MG recommend that corticosteroid dose is gradually tapered once treatment goals have been achieved.^28^ Despite the absence of protocol guidance on corticosteroid tapering, over one-third of patients on corticosteroids at baseline who had received ⩾2 consecutive symptom-driven cycles decreased or discontinued use whilst receiving cyclic rozanolixizumab treatment. Consistent improvements from baseline in MG-specific outcomes were observed across cycles, inclusive of patients who underwent corticosteroid dose tapering. At the population level, MG-ADL and QMG responses were maintained throughout repeated treatment cycles for up to 130 weeks.

Fatigue is a common patient-reported symptom of MG that negatively impacts patients’ daily activities and quality of life.^29,30^ However, due to its subjective and fluctuating nature, fatigue can be challenging to evaluate.^29^ Established MG-specific assessments, such as MG-ADL, QMG and MGC scores, do not assess fatigue as a standalone symptom, and evidence-based guidance on its management is limited.^30,31^ Here, inclusion of the MG Symptoms PRO, a novel PRO measure in MG, facilitated granular assessment of patients’ experiences of relevant MG symptoms, including physical fatigue.^31^ Consistent improvements from baseline to Day 43 in MG Symptoms PRO scale scores were demonstrated; the proportion of patients achieving the responder cut-offs of ⩾16.67 points for Muscle Weakness Fatigability and ⩾20.00 points for Physical Fatigue and Bulbar Muscle Weakness^32^ was broadly consistent within each scale across repeated cycles up to Cycle 11, with variability thereafter.

Chronic diseases such as MG often require treatment over extended periods, resulting in long-term patient exposure.^2,27^ Long-term safety follow-up allows for the observation of adverse events that may only manifest after prolonged exposure.^33^ Rozanolixizumab has demonstrated an acceptable safety profile during chronic weekly treatment for up to 52 weeks in the MG0004 study,^17^ with no significant increase in safety signals compared with one 6-week cycle in the MycarinG study and repeated treatment cycles in the interim analysis of MG0007.^9,19^ The safety profile observed across 223.8 total patient-years at risk in this final pooled analysis was consistent with the previously reported interim data.^19^ In general, incidence of TEAEs remained stable with repeated cyclic treatment among patients who remained in the study at each cycle, and total incidence was lower in the 7 mg/kg group than in the 10 mg/kg group. The most frequently reported TEAE, headache, is commonly associated with other targeted therapies for the treatment of gMG.^34,35^ The incidence of any headache (including migraine and migraine with aura) did not increase with repeated treatment cycles; most events were mild or moderate.

Patients receiving FcRn inhibitors may have increased susceptibility to infections due to the mechanism of action of such therapies.^36^ In line with other approved FcRn inhibitors, no substantial increase in serious infections was observed with repeated rozanolixizumab treatment, despite reduction in IgG serum concentration.^37^ The selective nature of FcRn inhibition, specifically its lack of impact on levels of other Ig isotypes such as IgA and IgM, may provide an explanation for the absence of reported increases in serious infections with FcRn inhibitors.^36,37^ Most infections observed during rozanolixizumab treatment were non-serious and mild or moderate in intensity; serious and severe infections were predominantly related to COVID-19.

Rozanolixizumab blocks FcRn at a site distinct to the albumin binding site, thus minimising the impact on serum albumin concentrations.^5,38^ In line with the mechanism of action of rozanolixizumab, a rapid, sustained and selective reduction in total IgG serum concentration was observed across cycles, with no clinically meaningful reduction in albumin or lipid levels recorded. These data suggest that whilst rozanolixizumab has a strong impact on total IgG serum concentration, there is not a significant effect on lipid or albumin metabolism.

The observed inter-patient variation in the duration of treatment-free intervals between symptom-driven cycles supports the usefulness of a personalised approach to treatment in gMG. Based on these data, patients received an average of approximately four treatment cycles in their first year of treatment, which equates to treatment-free intervals of 6–8 weeks between cycles. Hence, treatment with rozanolixizumab, given in 6-week cycles that are guided by a neurologist’s assessment of symptom worsening, offers a flexible approach that allows patients to personalise their therapy based on their symptoms.

In MycarinG, MG0004 and MG0007, rozanolixizumab was primarily administered by healthcare professionals (HCPs) via an infusion pump. However, eight patients in MG0007 received HCP-administered rozanolixizumab via the manual push method where the syringe plunger is pushed by hand. Manual push is an alternate method of administration that provides flexibility, could reduce infusion times versus administration via an infusion pump, and also facilitates self-administration.^39,40^ The safety and efficacy of rozanolixizumab was consistent following the switch from HCP-administered treatment using an infusion pump to HCP-administered treatment via the manual push method in these eight patients. To explore self-administration of rozanolixizumab using both infusion pump and manual push methods, the MG0020 study (NCT05681715) was conducted.^41^ Self-administered rozanolixizumab treatment via both methods after training from an HCP was subsequently approved by the European Medicines Agency,^12^ Japan’s Pharmaceuticals and Medical Devices Agency,^13^ and other regulatory bodies.

Limitations of this analysis include the open-label nature of the MG0004 and MG0007 studies, meaning that there was no placebo control, and the permitting of concomitant corticosteroid and NSIST dose changes during these OLE studies. Both the absence of a concurrent control group and the investigators’ discretionary modification of concomitant medications introduce confounding factors that were not adjusted for in the efficacy analyses. These factors make it difficult to separate the efficacy attributed to rozanolixizumab from regression to the mean, effects resulting from patients’ expectations of receiving an active treatment or the effects of permitted concomitant interventions. Whilst we cannot rule out a ‘placebo effect’, the absence of a control group is an inherent limitation of OLE studies. It should be noted that allowing investigators the discretion to modify concomitant corticosteroid and NSIST doses enabled patients to increase, decrease or discontinue these medications as clinically appropriate. This approach better reflects real-world clinical management than a protocol that prohibits such adjustments. Second, the primary efficacy pool comprised patients who had received ⩾2 symptom-driven rozanolixizumab treatment cycles; however, not all rozanolixizumab treatment cycles were symptom driven (e.g., patients who did not require rescue therapy upon entering MG0004 or MG0007), which reduced the number of patients, and therefore the number of cycles included in the efficacy analysis compared with the safety analysis. Due to small patient numbers in later cycles, the presentation of efficacy data was limited to 13 cycles. Lastly, there is a potential for attrition bias, as patients who did not respond to rozanolixizumab were more likely to have discontinued the study, enriching the remaining population with rozanolixizumab responders; and, per protocol, patients who received rescue therapy in response to MG worsening in MG0007 had to discontinue the study. Most discontinuations due to TEAEs occurred early in the study during Cycles 1 and 2, which may have further compounded this bias.

Pooling studies with differing designs and cycle initiation rules introduces structural heterogeneity, which should be considered when making conclusions of consistent, long-term efficacy. Additionally, attrition bias, caused by rozanolixizumab responders being more likely to remain in the study across repeated treatment cycles, might cause an overestimation of treatment effects. A mixed model for repeated measures was developed to assess study-stratified effects and the degree to which attrition bias may have resulted in an overestimation of efficacy. Study and cycle were fixed effects, with intercept and patient as random effects, for MG-ADL and QMG score changes (Cycles 1–13). Neither study nor cycle effect was statistically significant.

In light of the newer targeted therapies that have been approved for the treatment of gMG in recent years,^42^ an update to the international consensus guidance for the management of MG is required.^43,44^ The German guidelines for the management of myasthenic syndromes, which were updated in November 2024, suggest that inhibitors of FcRn, such as rozanolixizumab, will significantly change the MG treatment landscape.^45^ Similar sentiments were echoed in the British and Israeli guidelines for MG management, both of which were published in August 2025.^26,46^ These analyses of final data from the MycarinG, MG0004 and MG0007 studies provide insights into the long-term efficacy and safety of rozanolixizumab, which are important given the chronic and recurring nature of MG symptoms. Beyond the clinical trial data presented here, real-world data are needed to further establish the efficacy and safety of rozanolixizumab in clinical practice.

In conclusion, this integrated pooled analysis supports the efficacy and safety of repeated 6-week treatment cycles with rozanolixizumab in patients with anti-AChR Ab+ and anti-MuSK Ab+ gMG. The clinical efficacy of repeated treatment cycles with rozanolixizumab was demonstrated through consistent, rapid improvements across all efficacy endpoints, building on the positive results of the MycarinG study. Data indicate that rozanolixizumab has an acceptable safety profile and is generally well tolerated, providing further evidence for the use of rozanolixizumab as a potential option for the long-term treatment of gMG.

## Supplemental Material

sj-docx-1-tan-10.1177_17562864261458532 – Supplemental material for Long-term use of rozanolixizumab in generalised myasthenia gravis: final pooled analysis of the phase III MycarinG study and two open-label extensions Supplemental material, sj-docx-1-tan-10.1177_17562864261458532 for Long-term use of rozanolixizumab in generalised myasthenia gravis: final pooled analysis of the phase III MycarinG study and two open-label extensions by Vera Bril, Artur Drużdż, Julian Grosskreutz, Ali A. Habib, Renato Mantegazza, Sabrina Sacconi, Kimiaki Utsugisawa, Tuan Vu, Marion Boehnlein, Fiona Grimson, Niamh Houston, Virginie Kerbusch, Irene Pulido-Valdeolivas, Thaïs Tarancón and John Vissing in Therapeutic Advances in Neurological Disorders

## Declarations

**Ethics approval and consent to participate:** The MycarinG, MG0004 and MG0007 studies were carried out in compliance with local regulations, the International Council for Harmonisation Good Clinical Practice guidelines and the principles of the Declaration of Helsinki. The study protocol, amendments and patients’ informed consent were reviewed by a national, regional or independent ethics committee or institutional review board for each participating site. The approving ethics committees or institutional review boards are listed in the Supplemental Material. All patients provided written informed consent.

**Consent for publication:** Not applicable.

**Availability of data and materials:** Underlying data from this manuscript may be requested by qualified researchers 6 months after product approval in the USA and/or Europe, or global development is discontinued, and 18 months after trial completion. Investigators may request access to anonymised individual patient-level data and redacted trial documents which may include analysis-ready datasets, study protocol, annotated case report form, statistical analysis plan, dataset specifications and clinical study report. Prior to use of the data, proposals need to be approved by an independent review panel at www.Vivli.org and a signed data sharing agreement will need to be executed. All documents are available in English only, for a pre-specified time, typically 12 months, on a password-protected portal.
