# Supplementary material for: Long-term use of rozanolixizumab in generalised myasthenia gravis: final pooled analysis of the phase III MycarinG study and two open-label extensions
Source: Ther Adv Neurol Disord. 2026 Jun 29;19:17562864261458532. doi: 10.1177/17562864261458532 (PMC13319577; doi:10.1177/17562864261458532)
Supplement: sj-docx-1-tan-10.1177_17562864261458532 – Supplemental material for Long-term use of rozanolixizumab in generalised myasthenia gravis: final pooled analysis of the phase III MycarinG study and two open-label extensions [file sj-docx-1-tan-10.1177_17562864261458532.docx]

**Supplemental Material**

**Long-term use of rozanolixizumab in generalised myasthenia gravis: Final pooled analysis of the Phase 3 MycarinG study and two open-label extensions**

Vera Bril^1^, Artur Drużdż^2^, Julian Grosskreutz^3^, Ali A. Habib^4^, Renato Mantegazza^5^, Sabrina Sacconi^6^, Kimiaki Utsugisawa^7^, Tuan Vu^8^, Marion Boehnlein^9^, Fiona Grimson^10^, Niamh Houston^10^, Virginie Kerbusch^11^, Irene Pulido-Valdeolivas^12^, Thaïs Tarancón^12^, John Vissing^13^ on behalf of the MycarinG, MG0004 and MG0007 study investigators

^1^Ellen and Martin Prosserman Centre for Neuromuscular Diseases, Toronto General Hospital, University of Toronto, Toronto, Ontario, Canada; ^2^Department of Neurology, Municipal Hospital, Poznań, Poland; ^3^Precision Neurology of Neuromuscular Diseases, Department of Neurology, University of Lübeck, Lübeck, Germany; ^4^MDA ALS & Neuromuscular Center, Department of Neurology, University of California, Irvine, Orange, CA, USA; ^5^Emeritus and Past Director, Department of Neuroimmunology and Neuromuscular Diseases, Fondazione IRCCS, Istituto Nazionale Neurologico Carlo Besta, Milan, Italy; ^6^Université Côte d’Azur, Peripheral Nervous System and Muscle Department, Pasteur 2 Hospital, Centre Hospitalier Universitaire de Nice, Nice, France; ^7^Department of Neurology, Hanamaki General Hospital, Hanamaki, Japan; ^8^Department of Neurology, University of South Florida Morsani College of Medicine, Tampa, FL, USA; ^9^UCB, Monheim, Germany; ^10^UCB, Slough, UK; ^11^PharmAspire BV, Wijchen, Netherlands; ^12^UCB, Madrid, Spain; ^13^Copenhagen Neuromuscular Center, Department of Neurology, Rigshospitalet, University of Copenhagen, Copenhagen, Denmark

**Corresponding author:** Dr Vera Bril
**Email:** [vera.bril@utoronto.ca](mailto:vera.bril@utoronto.ca)

**Table of contents**

[Supplementary Table 1. Pool definitions 3](#_Toc217397128)

[Supplementary Table 2. Patient demographics and baseline characteristics
(primary safety pool) 4](#_Toc217397160)

[Supplementary Figure 1. Mean change from baseline to Day 43 in (a) MG-ADL, (b) MGC,
(c) QMG, (d) MG Symptoms PRO Muscle Weakness Fatigability, (e) MG Symptoms PRO Physical Fatigue and (f) MG Symptoms PRO Bulbar Muscle Weakness scores by treatment group 5](#_Toc217397161)

[Supplementary Figure 2. Responder rates at Day 43 during each cycle for MG Symptoms PRO (a) Muscle Weakness Fatigability, (b) Physical Fatigue and (c) Bulbar Muscle Weakness scores 8](#_Toc217397162)

[Supplementary Table 3. Responder rates at Day 43 during each cycle for MG-ADL, MGC, QMG and MG Symptoms PRO Muscle Weakness Fatigability, Physical Fatigue and Bulbar Muscle Weakness scores by treatment group 9](#_Toc217397163)

[Supplementary Table 4. Achievement of MSE by treatment group and overall 11](#_Toc217397164)

[Supplementary Table 5. Overview of TEAEs by treatment group 12](#_Toc217397165)

[Supplementary Table 6. MycarinG, MG0004 and MG0007 co-investigators
and contributors 16](#_Toc217397166)

[Institutional Review Boards and Independent Ethics Committees 31](#_Toc217397167)

## Supplementary Table 1. Pool definitions

| Pool and purpose | Definition | Studies included | Patients, N |
| --- | --- | --- | --- |
| Primary efficacy pool: To assess response to repeated symptom-driven cyclical treatment | Patients who had received ≥2 symptom-driven treatment cycles | MycarinG (rozanolixizumab data only)  MG0004 (first 6 weeks only for patients who required rescue therapy in the observation period of MycarinG)  MG0007 (symptom-driven cycles only) | 129 |
| Pool E2: To assess treatment-free intervals | Patients who had received rozanolixizumab treatment and had initiated or were awaiting a symptom-driven treatment cycle | MycarinG (rozanolixizumab data only)  MG0004 (first 6 weeks only for patients who required rescue therapy in the observation period of MycarinG)  MG0007 (symptom-driven cycles only) | 167 |
| Pool E3: To assess response to repeated symptom-driven cyclical treatment with no chronic weekly or fixed treatment cycles | Patients who had received ≥2 consecutive symptom-driven treatment cycles | MycarinG (rozanolixizumab data only)  MG0007 (symptom-driven cycles only) | 121 |
| Primary safety pool: To assess the safety of rozanolixizumab treatment cycles | Patients who had received ≥1 dose of rozanolixizumab in any 6-week treatment period followed by an up to 8-week follow-up period after the last infusion | MycarinG (rozanolixizumab data only)  MG0007 | 188 |
| Immunogenicity pool: To assess the immunogenicity of rozanolixizumab | Patients who had received only cyclic treatment | MycarinG (rozanolixizumab data only)  MG0007 (excluding data from patients who previously participated in MG0004) | 168 |

## Supplementary Table 2. Patient demographics and baseline characteristics (primary safety pool)

|  | |  | **RLZ 7 mg/kg** (N=94) | **RLZ 10 mg/kg**  (N=94) | **RLZ total** (N=188) |
| --- | --- | --- | --- | --- | --- |
| Age, years, mean (SD) | | | 53.1 (14.9) | 52.0 (17.6) | 52.5 (16.3) |
| Sex, female, n (%) | | | 56 (59.6) | 55 (58.5) | 111 (59.0) |
| **Body weight,  n (%)** | | <50 kg | 9 (9.6) | 2 (2.1) | 11 (5.9) |
|  |  | 50 to <70 kg | 22 (23.4) | 37 (39.4) | 59 (31.4) |
|  |  | 70 to <100 kg | 44 (46.8) | 32 (34.0) | 76 (40.4) |
|  |  | ≥100 kg | 19 (20.2) | 23 (24.5) | 42 (22.3) |
| BMI, kg/m^2^, mean (SD) | | | 27.5 (6.5) | 28.1 (6.4) | 27.8 (6.5) |
| **Geographic region, n (%)** | | North America | 30 (31.9) | 24 (25.5) | 54 (28.7) |
|  |  | Europe | 52 (55.3) | 62 (66.0) | 114 (60.6) |
|  |  | Asia (excl. Japan) | 4 (4.3) | 3 (3.2) | 7 (3.7) |
|  |  | Japan | 8 (8.5) | 5 (5.3) | 13 (6.9) |
| **Race, n (%)*** | | Asian | 12 (12.8) | 9 (9.6) | 21 (11.2) |
|  |  | Black | 0 | 4 (4.3) | 4 (2.1) |
|  |  | Native Hawaiian or other Pacific Islander | 0 | 1 (1.1) | 1 (0.5) |
|  |  | White | 58 (61.7) | 69 (73.4) | 127 (67.6) |
|  |  | Missing | 24 (25.5) | 11 (11.7) | 35 (18.6) |
| Age at initial gMG diagnosis, years, mean (SD) | | | 45.5 (16.2) | 43.2 (19.8) | 44.3 (18.0) |
| Duration of disease, years, mean (SD) | | | 7.9 (7.9) | 9.0 (9.2) | 8.5 (8.6) |
| **MGFA Disease Class, n (%)** | | II | 45 (47.9) | 30 (31.9) | 75 (39.9) |
|  |  | III | 45 (47.9) | 62 (66.0) | 107 (56.9) |
|  |  | IV | 4 (4.3) | 2 (2.1) | 6 (3.2) |
| MG crisis, n (%) | | | 25 (26.6) | 28 (29.8) | 53 (28.2) |
| Thymectomy, yes, n (%) | | | 43 (45.7) | 32 (34.0) | 75 (39.9) |
| Anti-AChR Ab+, n (%) | | | 84 (89.4) | 86 (91.5) | 170 (90.4) |
| Anti-MuSK Ab+, n (%) | | | 9 (9.6) | 9 (9.6) | 18 (9.6) |
| MG-ADL score, mean (SD) | | | 8.3 (3.7) | 8.4 (2.9) | 8.3 (3.4) |
| QMG score, mean (SD) | | | 15.4 (3.6) | 15.8 (3.6) | 15.6 (3.6) |
| Total IgG, g/L, mean (SD) | | | 10.2 (2.9) | 9.8 (2.7) | 10.0 (2.8) |
| No prior IVIg or PLEX, n (%) | | | 77 (81.9) | 73 (77.7) | 150 (79.8) |
| **Baseline gMG medication,  n (%)** | CS for systemic use | | 56 (59.6) | 64 (68.1) | 120 (63.8) |
|  | Immunosuppressants | | 45 (47.9) | 52 (55.3) | 97 (51.6) |
|  | Parasympathomimetics | | 80 (85.1) | 82 (87.2) | 162 (86.2) |

Primary safety pool. *Data on race were not permitted to be collected in France and Canada. Ab+, antibody positive; AChR, acetylcholine receptor; BMI, body mass index; CS, corticosteroid; gMG, generalised myasthenia gravis; IgG, immunoglobulin G; IVIg, intravenous immunoglobulin; MG, myasthenia gravis; MG-ADL, Myasthenia Gravis Activities of Daily Living; MGFA, Myasthenia Gravis Foundation of America; MuSK, muscle-specific tyrosine kinase; PLEX, plasma exchange; QMG, Quantitative Myasthenia Gravis; RLZ, rozanolixizumab; SD, standard deviation.

## Supplementary Figure 1. Mean change from baseline to Day 43 in (a) MG-ADL, (b) MGC, (c) QMG, (d) MG Symptoms PRO Muscle Weakness Fatigability, (e) MG Symptoms PRO Physical Fatigue and (f) MG Symptoms PRO Bulbar Muscle Weakness scores by treatment group


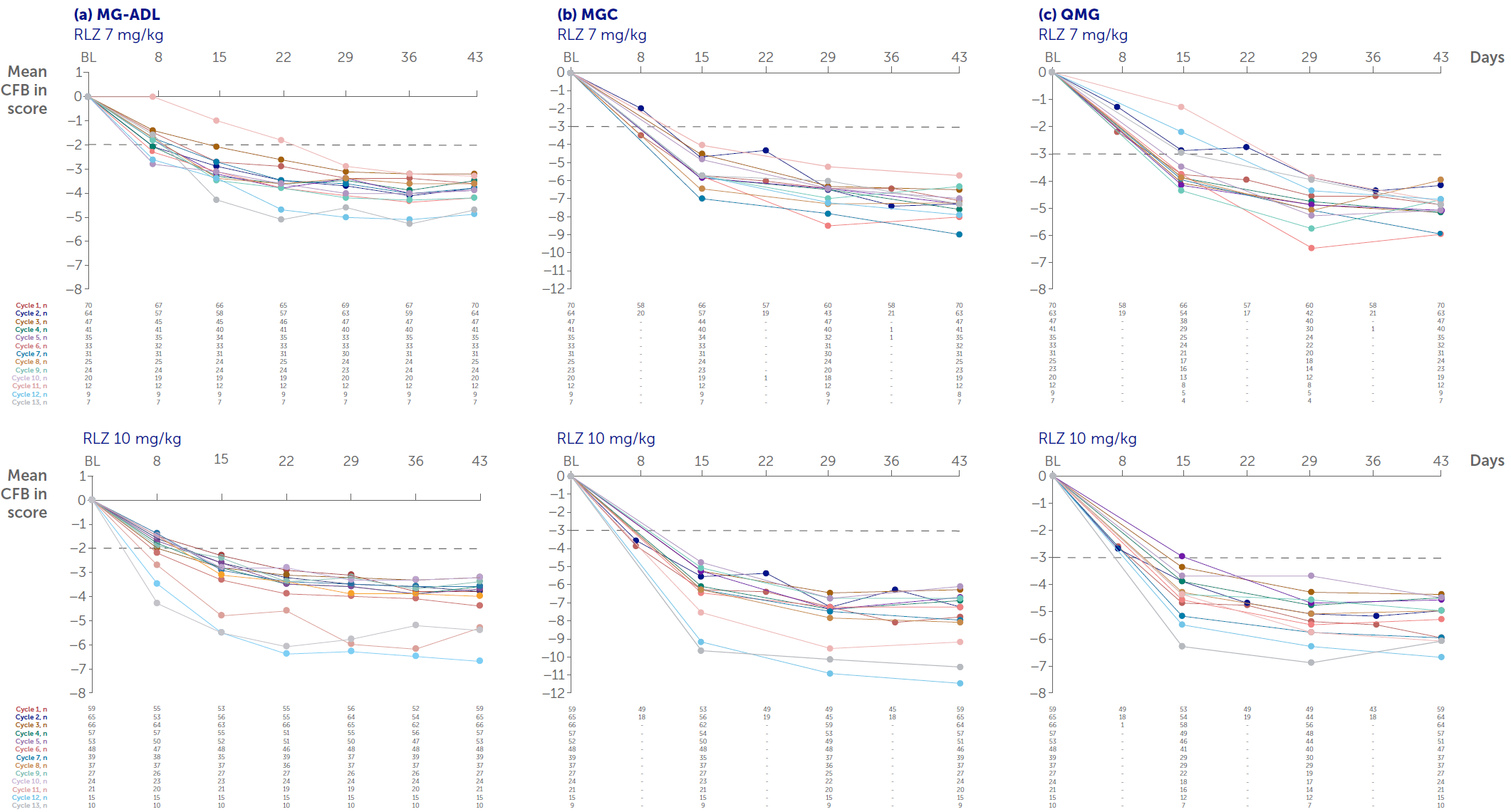


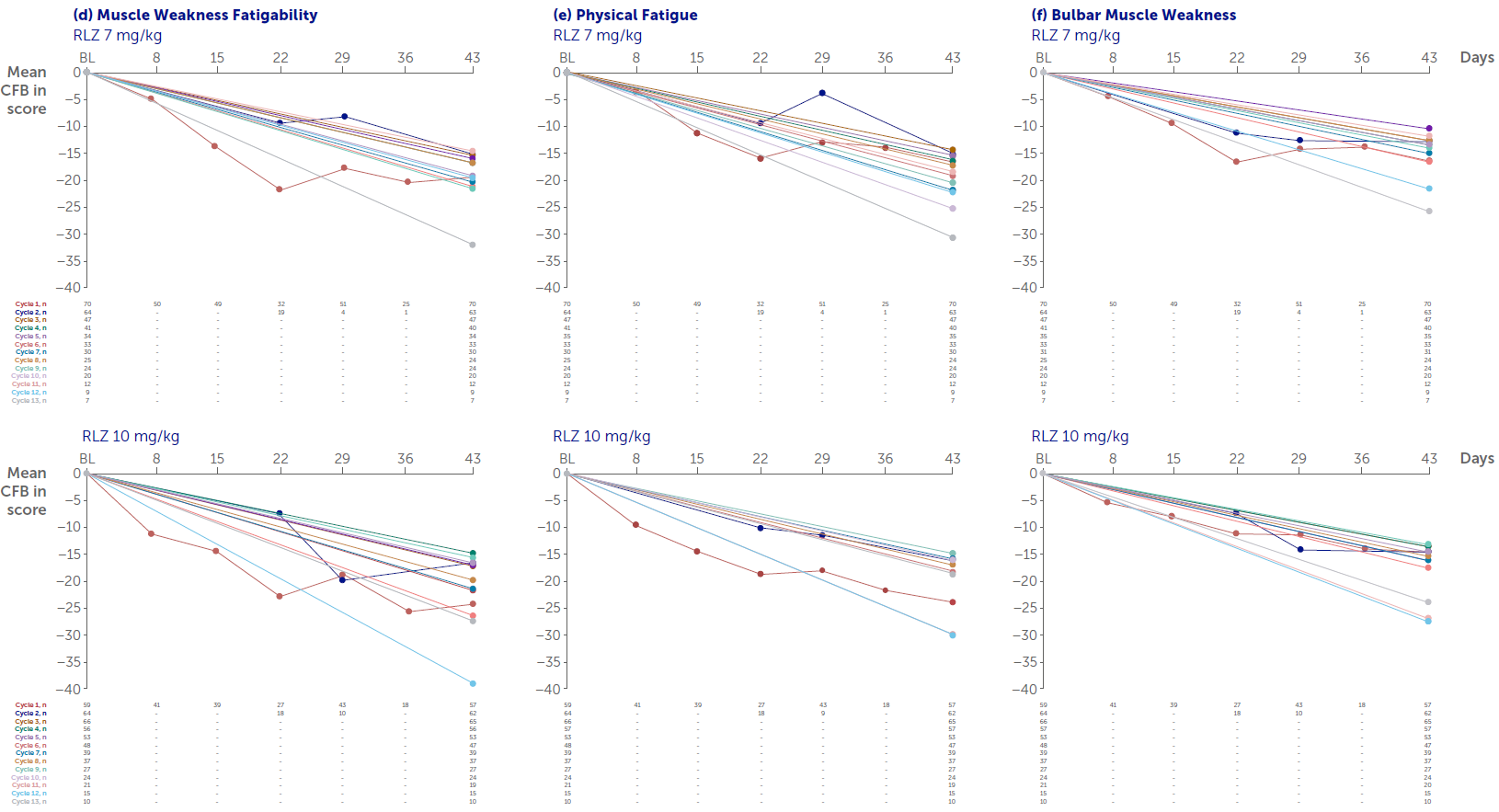


Primary efficacy pool. Efficacy data collected at or after the time point of rescue use were excluded from the analysis with no imputation of missing data for the respective cycle. The reference values for meaningful change were a 2-point improvement for MG-ADL and a 3-point improvement for MGC and QMG. The reference values for meaningful within-patient change for the MG Symptoms PRO scales were a 16.67-point improvement for Muscle Weakness Fatigability and a 20.00-point improvement for Physical Fatigue and Bulbar Muscle Weakness. BL, baseline; CFB, change from baseline; MG-ADL, Myasthenia Gravis Activities of Daily Living; MGC, Myasthenia Gravis Composite; MG Symptoms PRO, Myasthenia Gravis Symptoms Patient-Reported Outcome; QMG, Quantitative Myasthenia Gravis; RLZ, rozanolixizumab.

## Supplementary Figure 2. Responder rates at Day 43 during each cycle for MG Symptoms PRO (a) Muscle Weakness Fatigability, (b) Physical Fatigue and (c) Bulbar Muscle Weakness scores


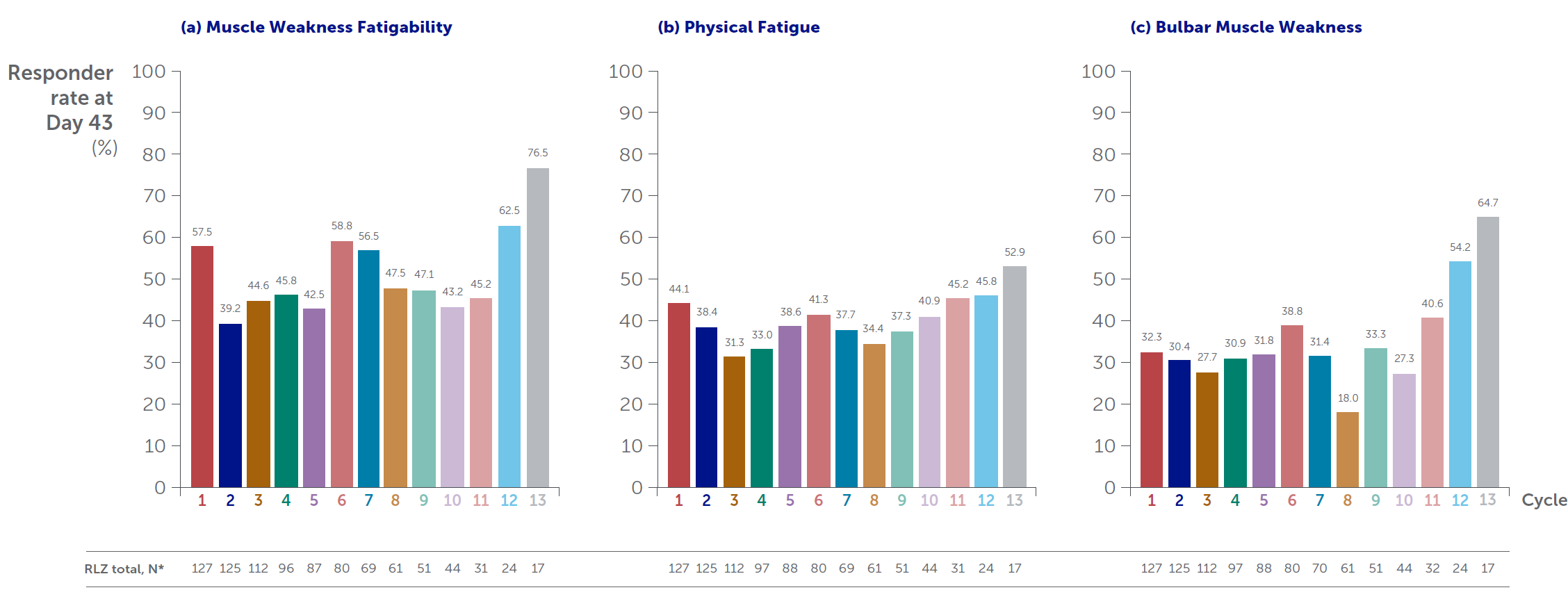


Primary efficacy pool. MG Symptoms PRO Muscle Weakness Fatigability, Physical Fatigue and Bulbar Muscle Weakness response were defined as a
≥16.67-point, ≥20.00-point and ≥20.00-point within-patient improvement in score from baseline, respectively. Patients with missing data at Day 43 or who received rescue therapy after Day 43 were treated as missing. *N represents the number of patients who had completed the relevant MG Symptoms PRO scale assessment at Day 43 in each treatment cycle. MG Symptoms PRO, Myasthenia Gravis Symptoms Patient-Reported Outcome; RLZ, rozanolixizumab.

## Supplementary Table 3. Responder rates at Day 43 during each cycle for MG-ADL, MGC, QMG and MG Symptoms PRO Muscle Weakness Fatigability, Physical Fatigue and Bulbar Muscle Weakness scores by treatment group

| **Cycle** | **RLZ dose** |  | |  | |  | **MG Symptoms PRO^‡^** | | |
| --- | --- | --- | --- | --- | --- | --- | --- | --- | --- |
|  |  | **MG-ADL,  n/Nsub (%)*** | **MGC,  n/Nsub (%)^†^** | | **QMG,  n/Nsub (%)^†^** | | **Muscle Weakness Fatigability, n/Nsub (%)** | **Physical Fatigue,  n/Nsub (%)** | **Bulbar Muscle Weakness,  n/Nsub (%)** |
| **Cycle 1** | **7 mg/kg** | 53/70 (75.7) | 49/70 (70.0) | | 43/70 (61.4) | | 37/70 (52.9) | 24/70 (34.3) | 22/70 (31.4) |
|  | **10 mg/kg** | 43/59 (72.9) | 46/59 (78.0) | | 45/59 (76.3) | | 36/57 (63.2) | 32/57 (56.1) | 19/57 (33.3) |
| **Cycle 2** | **7 mg/kg** | 50/64 (78.1) | 46/64 (71.9) | | 34/63 (54.0) | | 21/63 (33.3) | 23/63 (36.5) | 17/63 (27.0) |
|  | **10 mg/kg** | 46/65 (70.8) | 50/65 (76.9) | | 44/64 (68.8) | | 28/62 (45.2) | 25/62 (40.3) | 21/62 (33.9) |
| **Cycle 3** | **7 mg/kg** | 31/47 (66.0) | 32/47 (68.1) | | 33/47 (70.2) | | 21/47 (44.7) | 13/47 (27.7) | 14/47 (29.8) |
|  | **10 mg/kg** | 41/66 (62.1) | 45/65 (69.2) | | 41/64 (64.1) | | 29/65 (44.6) | 22/65 (33.8) | 17/65 (26.2) |
| **Cycle 4** | **7 mg/kg** | 31/41 (75.6) | 33/41 (80.5) | | 28/40 (70.0) | | 20/40 (50.0) | 14/40 (35.0) | 13/40 (32.5) |
|  | **10 mg/kg** | 40/57 (70.2) | 40/57 (70.2) | | 34/57 (59.6) | | 24/56 (42.9) | 18/57 (31.6) | 17/57 (29.8) |
| **Cycle 5** | **7 mg/kg** | 32/35 (91.4) | 27/35 (77.1) | | 21/35 (60.0) | | 15/34 (44.1) | 14/35 (40.0) | 8/35 (22.9) |
|  | **10 mg/kg** | 38/53 (71.7) | 36/52 (69.2) | | 33/51 (64.7) | | 22/53 (41.5) | 20/53 (37.7) | 20/53 (37.7) |
| **Cycle 6** | **7 mg/kg** | 25/33 (75.8) | 24/32 (75.0) | | 22/32 (68.8) | | 19/33 (57.6) | 14/33 (42.4) | 13/33 (39.4) |
|  | **10 mg/kg** | 37/48 (77.1) | 37/46 (80.4) | | 33/47 (70.2) | | 28/47 (59.6) | 19/47 (40.4) | 18/47 (38.3) |
| **Cycle 7** | **7 mg/kg** | 24/31 (77.4) | 26/31 (83.9) | | 25/31 (80.6) | | 17/30 (56.7) | 13/30 (43.3) | 9/31 (29.0) |
|  | **10 mg/kg** | 30/39 (76.9) | 35/39 (89.7) | | 30/38 (78.9) | | 22/39 (56.4) | 13/39 (33.3) | 13/39 (33.3) |
| **Cycle 8** | **7 mg/kg** | 18/25 (72.0) | 20/25 (80.0) | | 17/24 (70.8) | | 11/24 (45.8) | 7/24 (29.2) | 4/24 (16.7) |
|  | **10 mg/kg** | 29/37 (78.4) | 32/37 (86.5) | | 24/37 (64.9) | | 18/37 (48.6) | 14/37 (37.8) | 7/37 (18.9) |
| **Cycle 9** | **7 mg/kg** | 16/24 (66.7) | 17/24 (70.8) | | 13/24 (54.2) | | 12/24 (50.0) | 11/24 (45.8) | 10/24 (41.7) |
|  | **10 mg/kg** | 19/27 (70.4) | 21/27 (77.8) | | 18/27 (66.7) | | 12/27 (44.4) | 8/27 (29.6) | 7/27 (25.9) |
| **Cycle 10** | **7 mg/kg** | 15/20 (75.0) | 13/19 (68.4) | | 15/19 (78.9) | | 11/20 (55.0) | 10/20 (50.0) | 7/20 (35.0) |
|  | **10 mg/kg** | 16/24 (66.7) | 19/24 (79.2) | | 16/24 (66.7) | | 8/24 (33.3) | 8/24 (33.3) | 5/24 (20.8) |
| **Cycle 11** | **7 mg/kg** | 7/12 (58.3) | 8/12 (66.7) | | 7/12 (58.3) | | 4/12 (33.3) | 5/12 (41.7) | 4/12 (33.3) |
|  | **10 mg/kg** | 19/21 (90.5) | 17/21 (81.0) | | 13/21 (61.9) | | 10/19 (52.6) | 9/19 (47.4) | 9/20 (45.0) |
| **Cycle 12** | **7 mg/kg** | 7/9 (77.8) | 7/9 (77.8) | | 6/9 (66.7) | | 4/9 (44.4) | 4/9 (44.4) | 5/9 (55.6) |
|  | **10 mg/kg** | 13/15 (86.7) | 13/15 (86.7) | | 13/15 (86.7) | | 11/15 (73.3) | 7/15 (46.7) | 8/15 (53.3) |
| **Cycle 13** | **7 mg/kg** | 7/7 (100) | 6/7 (85.7) | | 5/7 (71.4) | | 7/7 (100) | 5/7 (71.4) | 5/7 (71.4) |
|  | **10 mg/kg** | 8/10 (80.0) | 8/10 (80.0) | | 9/10 (90.0) | | 6/10 (60.0) | 4/10 (40.0) | 6/10 (60.0) |

Primary efficacy pool. Observed data. *≥2-point improvement without rescue therapy. ^†^≥3-point improvement without rescue therapy. ^‡^MG Symptoms PRO Muscle Weakness Fatigability, Physical Fatigue and Bulbar Muscle Weakness response were defined as a ≥16.67-point, ≥20.00-point and ≥20.00-point within-patient improvement in score from baseline, respectively. Patients with missing data at Day 43 or who received rescue therapy after Day 43 were treated as missing. MG-ADL, Myasthenia Gravis Activities of Daily Living; MGC, Myasthenia Gravis Composite; MG Symptoms PRO, Myasthenia Gravis Symptoms Patient-Reported Outcome; QMG, Quantitative Myasthenia Gravis; RLZ, rozanolixizumab.

## Supplementary Table 4. Achievement of MSE by treatment group and overall

| **Cycle** | **Achievement of MSE, n/Nsub (%)** | | |
| --- | --- | --- | --- |
|  | **RLZ 7 mg/kg** | **RLZ 10 mg/kg** | **RLZ total** |
| **Cycle 1** | 18/70 (25.7) | 18/59 (30.5) | 36/129 (27.9) |
| **Cycle 2** | 21/64 (32.8) | 14/65 (21.5) | 35/129 (27.1) |
| **Cycle 3** | 15/47 (31.9) | 15/66 (22.7) | 30/113 (26.5) |
| **Cycle 4** | 15/41 (36.6) | 14/57 (24.6) | 29/98 (29.6) |
| **Cycle 5** | 15/35 (42.9) | 11/53 (20.8) | 26/88 (29.5) |
| **Cycle 6** | 13/33 (39.4) | 13/48 (27.1) | 26/81 (32.1) |
| **Cycle 7** | 14/31 (45.2) | 11/39 (28.2) | 25/70 (35.7) |
| **Cycle 8** | 11/25 (44.0) | 10/37 (27.0) | 21/62 (33.9) |
| **Cycle 9** | 9/24 (37.5) | 11/27 (40.7) | 20/51 (39.2) |
| **Cycle 10** | 8/20 (40.0) | 9/24 (37.5) | 17/44 (38.6) |
| **Cycle 11** | 3/12 (25.0) | 9/21 (42.9) | 12/33 (36.4) |
| **Cycle 12** | 1/9 (11.1) | 8/15 (53.3) | 9/24 (37.5) |
| **Cycle 13** | 2/7 (28.6) | 6/10 (60.0) | 8/17 (47.1) |

Primary efficacy pool. MSE was defined as an MG-ADL score or 0 or 1 at any visit for each 6-week cycle and follow-up period without the use of rescue therapy. MG-ADL, Myasthenia Gravis Activities of Daily Living; MSE, minimal symptom expression; RLZ, rozanolixizumab.

## Supplementary Table 5. Overview of TEAEs by treatment group

| **n (%)** | **All cycles^†^** | | **Cycle 1** | | **Cycle 2** | | **Cycle 3** | | **Cycle 4** | | **Cycle 5** | | **Cycle 6** | |
| --- | --- | --- | --- | --- | --- | --- | --- | --- | --- | --- | --- | --- | --- | --- |
|  | **RLZ 7 mg/kg**  **N=135** | **RLZ 10 mg/kg**  **N=133** | **RLZ 7 mg/kg**  **N=94** | **RLZ 10 mg/kg**  **N=94** | **RLZ 7 mg/kg**  **N=73** | **RLZ 10 mg/kg**  **N=72** | **RLZ 7 mg/kg**  **N=51** | **RLZ 10 mg/kg**  **N=66** | **RLZ 7 mg/kg**  **N=43** | **RLZ 10 mg/kg**  **N=59** | **RLZ 7 mg/kg**  **N=40** | **RLZ 10 mg/kg**  **N=54** | **RLZ 7 mg/kg**  **N=35** | **RLZ 10 mg/kg**  **N=51** |
| **Any TEAEs*** | 112 (83.0) | 126 (94.7) | 70 (74.5) | 79 (84.0) | 47 (64.4) | 57 (79.2) | 30 (58.8) | 46 (69.7) | 24 (55.8) | 41 (69.5) | 22 (55.0) | 48 (88.9) | 23 (65.7) | 40 (78.4) |
| Headache | 58 (43.0) | 61 (45.9) | 35 (37.2) | 34 (36.2) | 17 (23.3) | 17 (23.6) | 5  (9.8) | 16 (24.2) | 7  (16.3) | 11 (18.6) | 3  (7.5) | 11 (20.4) | 7  (20.0) | 8  (15.7) |
| Diarrhoea | 35 (25.9) | 36 (27.1) | 20 (21.3) | 16 (17.0) | 6 (8.2) | 5 (6.9) | 1 (2.0) | 6 (9.1) | 7 (16.3) | 3 (5.1) | 5 (12.5) | 6 (11.1) | 4 (11.4) | 6 (11.8) |
| COVID-19 | 16 (11.9) | 26 (19.5) | 3 (3.2) | 1 (1.1) | 5 (6.8) | 6 (8.3) | 3 (5.9) | 3 (4.5) | - | 5 (8.5) | 2 (5.0) | 1 (1.9) | 1 (2.9) | 5 (9.8) |
| Pyrexia | 16 (11.9) | 28 (21.1) | 8  (8.5) | 17 (18.1) | 5  (6.8) | 4  (5.6) | 1  (2.0) | 1  (1.5) | 2  (4.7) | 3  (5.1) | 2  (5.0) | - | 1  (2.9) | 4  (7.8) |
| Nausea | 14 (10.4) | 23 (17.3) | 6 (6.4) | 9 (9.6) | 6 (8.2) | 3 (4.2) | 1 (2.0) | 5 (7.6) | 1 (2.3) | 3 (5.1) | 1 (2.5) | 4 (7.4) | 1 (2.9) | 1 (2.0) |
| MG worsening | 9  (6.7) | 19 (14.3) | 4 (4.3) | 5 (5.3) | 1 (1.4) | 1 (1.4) | 1 (2.0) | 1 (1.5) | 1 (2.3) | 1 (1.7) | 1 (2.5) | 4 (7.4) | 1 (2.9) | - |
| Arthralgia | 9  (6.7) | 15 (11.3) | 5 (5.3) | 5 (5.3) | 1 (1.4) | 3 (4.2) | 1 (2.0) | 2 (3.0) | 2 (4.7) | 4 (6.8) | - | 1 (1.9) | 1 (2.9) | 1 (2.0) |
| Nasopharyngitis | 11  (8.1) | 14 (10.5) | 2 (2.1) | 5 (5.3) | - | - | 3 (5.9) | 2 (3.0) | 2 (4.7) | 2 (3.4) | 2 (5.0) | 1 (1.9) | 2 (5.7) | - |
| Decreased blood IgG | 6  (4.4) | 15 (11.3) | 2 (2.1) | 2 (2.1) | 3 (4.1) | 5 (6.9) | 1 (2.0) | 4 (6.1) | 2 (4.7) | 5 (8.5) | 2 (5.0) | 3 (5.6) | 1 (2.9) | 3 (5.9) |
| Abdominal pain | 10 (7.4) | 9 (6.8) | 2 (2.1) | 3 (3.2) | 3 (4.1) | 2 (2.8) | - | 2 (3.0) | 2 (4.7) | 1 (1.7) | - | - | 2 (5.7) | - |
| URTI | 9 (6.7) | 11 (8.3) | 2 (2.1) | 1 (1.1) | 2 (2.7) | 2 (2.8) | - | 1 (1.5) | 1 (2.3) | - | 1 (2.5) | 3 (5.6) | 1 (2.9) | 3 (5.9) |
| **Serious TEAEs^‡^** | 21 (15.6) | 36 (27.1) | 7  (7.4) | 13 (13.8) | 3  (4.1) | 6  (8.3) | 1  (2.0) | 4  (6.1) | 2  (4.7) | 4  (6.8) | 2  (5.0) | 6  (11.1) | 2  (5.7) | - |
| MG worsening | 6 (4.4) | 13 (9.8) | 2 (2.1) | 3 (3.2) | 1 (1.4) | 1 (1.4) | 1 (2.0) | 1 (1.5) | 0 | 1 (1.7) | 1 (2.5) | 2 (3.7) | - | - |
| MG crisis | - | 4 (3.0) | - | 1 (1.1) | - | 2 (2.8) | - | 1 (1.5) | - | - | - | - | - | - |
| **Permanent discontinuation from study due to TEAEs** | 11  (8.1) | 22 (16.5) | 3  (3.2) | 10 (10.6) | 3  (4.1) | 5  (6.9) | 1  (2.0) | 2  (3.0) | 1  (2.3) | 2  (3.4) | - | 1  (1.9) | - | - |
| **Treatment-related TEAEs** | 66 (48.9) | 84 (63.2) | 38 (40.4) | 56 (59.6) | 18 (24.7) | 33 (45.8) | 8  (15.7) | 18 (27.3) | 11 (25.6) | 21 (35.6) | 9  (22.5) | 21 (38.9) | 13 (37.1) | 19 (37.3) |
| **Severe TEAEs^‡^** | 18 (13.3) | 46 (34.6) | 4  (4.3) | 19 (20.2) | 2  (2.7) | 7  (9.7) | 1  (2.0) | 5  (7.6) | 3  (7.0) | 6  (10.2) | 2  (5.0) | 7  (13.0) | 4  (11.4) | 2  (3.9) |
| MG worsening | 5 (3.7) | 12 (9.0) | 2 (2.1) | 3 (3.2) | 1 (1.4) | - | 1 (2.0) | 1 (1.5) | - | 1 (1.7) | 1 (2.5) | 2 (3.7) | - | - |
| Headache | 1 (0.7) | 7 (5.3) | 1 (1.1) | 6 (6.4) | - | - | - | - | - | - | - | - | - | 1 (2.0) |
| MG crisis | - | 4 (3.0) | - | 1 (1.1) | - | 2 (2.8) | - | 1 (1.5) | - | - | - | - | - | - |
| Decreased blood IgG | 1 (0.7) | 3 (2.3) | - | - | - | - | - | 1 (1.5) | 1 (2.3) | 2 (3.4) | - | 1 (1.9) | - | - |
| **TEAEs leading to death** | 1 (0.7) | 3 (2.3) | - | - | 1 (1.4) | 1 (1.4) | - | 1 (1.5) | - | - | - | - | - | - |

| **n (%)** | **Cycle 7** | | **Cycle 8** | | **Cycle 9** | | **Cycle 10** | | **Cycle 11** | | **Cycle 12** | | **Cycle 13** | |
| --- | --- | --- | --- | --- | --- | --- | --- | --- | --- | --- | --- | --- | --- | --- |
|  | **RLZ 7 mg/kg**  **N=31** | **RLZ 10 mg/kg**  **N=47** | **RLZ 7 mg/kg**  **N=29** | **RLZ 10 mg/kg**  **N=42** | **RLZ 7 mg/kg**  **N=26** | **RLZ 10 mg/kg**  **N=33** | **RLZ 7 mg/kg**  **N=20** | **RLZ 10 mg/kg**  **N=30** | **RLZ 7 mg/kg**  **N=13** | **RLZ 10 mg/kg**  **N=24** | **RLZ 7 mg/kg**  **N=8** | **RLZ 10 mg/kg**  **N=18** | **RLZ 7 mg/kg**  **N=8** | **RLZ 10 mg/kg**  **N=10** |
| **Any TEAEs*** | 17 (54.8) | 35 (74.5) | 14 (48.3) | 28 (66.7) | 13 (50.0) | 21 (63.6) | 14 (70.0) | 17 (56.7) | 8  (61.5) | 15 (62.5) | 3  (37.5) | 8  (44.4) | 5  (62.5) | 6  (60.0) |
| Headache | 4  (12.9) | 12 (25.5) | 4 (13.8) | 6 (14.3) | 4 (15.4) | 9 (27.3) | 2 (10.0) | 5 (16.7) | 3 (23.1) | 2 (8.3) | - | - | 1 (12.5) | - |
| Diarrhoea | - | 4 (8.5) | 3 (10.3) | 4 (9.5) | 2 (7.7) | 2 (6.1) | 1 (5.0) | 2 (6.7) | 1 (7.7) | - | - | - | - | 1 (10.0) |
| COVID-19 | - | 3 (6.4) | 1 (3.4) | 1 (2.4) | - | 1 (3.0) | 1 (5.0) | - | - | 2 (8.3) | - | - | - | - |
| Pyrexia | 1 (3.2) | 3 (6.4) | 1 (3.4) | 2 (4.8) | 3 (11.5) | 1 (3.0) | 1 (5.0) | 2 (6.7) | - | 3 (12.5) | - | - | 1 (12.5) | 1 (10.0) |
| Nausea | - | 1 (2.1) | 1 (3.4) | 1 (2.4) | 1 (3.8) | 1 (3.0) | - | 1 (3.3) | 1 (7.7) | - | - | - | - | 1 (10.0) |
| MG worsening | 1 (3.2) | 1 (2.1) | - | 2 (4.8) | - | 1 (3.0) | - | 2 (6.7) | - | 1 (4.2) | - | 1 (5.6) | 1 (12.5) | 1 (10.0) |
| Arthralgia | - | - | - | 1 (2.4) | - | - | - | 1 (3.3) | - | - | - | - | - | - |
| Nasopharyngitis | 1 (3.2) | 1 (2.1) | 3 (10.3) | 3 (7.1) | - | 1 (3.0) | 1 (5.0) | 3 (10.0) | - | 1 (4.2) | - | - | - | - |
| Decreased blood IgG | - | 2 (4.3) | 1 (3.4) | 1 (2.4) | - | - | - | - | - | 1 (4.2) | - | - | - | - |
| Abdominal pain | - | - | 1 (3.4) | - | 1 (3.8) | 1 (3.0) | - | - | - | - | - | 1 (5.6) | - | - |
| URTI | 2 (6.5) | 2 (4.3) | 1 (3.4) | 1 (2.4) | 1 (3.8) | 1 (3.0) | 2 (10.0) | 2 (6.7) | 1 (7.7) | - | - | 2 (11.1) | - | 1 (10.0) |
| **Serious TEAEs^‡^** | 2 (6.5) | 3 (6.4) | 2 (6.9) | 3 (7.1) | - | 1 (3.0) | 1 (5.0) | 2 (6.7) | - | 1 (4.2) | - | 1 (5.6) | 1 (12.5) | 1 (10.0) |
| MG worsening | 1 (3.2) | - | - | 1 (2.4) | - | 1 (3.0) | - | 1 (3.3) | - | 1 (4.2) | - | 1 (5.6) | 1 (12.5) | 1 (10.0) |
| MG crisis | - | - | - | - | - | - | - | - | - | - | - | - | - | - |
| **Permanent discontinuation from study due to TEAEs** | - | 1 (2.1) | - | 1 (2.4) | - | - | 2 (10.0) | - | 1 (7.7) | - | - | - | - | - |
| **Treatment-related TEAEs** | 8  (25.8) | 16 (34.0) | 9  (31.0) | 12 (28.6) | 8  (30.8) | 10 (30.3) | 4  (20.0) | 10 (33.3) | 5  (38.5) | 7  (29.2) | 2  (25.0) | 2  (11.1) | 2  (25.0) | 2  (20.0) |
| **Severe TEAEs^‡^** | 1 (3.2) | 2 (4.3) | 2 (6.9) | 3 (7.1) | - | 3 (9.1) | 2 (10.0) | 3 (10.0) | - | 1 (4.2) | - | 1 (5.6) | 1 (12.5) | 1 (10.0) |
| MG worsening | 1 (3.2) | - | - | 1 (2.4) | - | 1 (3.0) | - | 1 (3.3) | - | 1 (4.2) | - | 1 (5.6) | 1 (12.5) | 1 (10.0) |
| Headache | - | - | - | - | - | 1 (3.0) | - | 1 (3.3) | - | - | - | - | - | - |
| MG crisis | - | - | - | - | - | - | - | - | - | - | - | - | - | - |
| Decreased blood IgG | - | - | - | - | - | - | - | - | - | - | - | - | - | - |
| **TEAEs leading to death** | - | 1 (2.1) | - | - | - | - | - | - | - | - | - | - | - | - |

Primary safety pool. n is the number of patients reporting ≥1 TEAE within the category and cycle. For data presented by dose, allocation of patients to treatment groups was according to the most recent dose received. *The individual TEAEs presented underneath ‘Any TEAEs’ include those reported for ≥10% of all patients in the ‘All cycles’ column. ^†^Sum of cycles (highest dose received) = 484 for RLZ 7 mg/kg and 610 for RLZ 10 mg/kg. Data past Cycle 13 may be included. ^‡^The individual TEAEs presented underneath ‘Serious TEAEs’ and ‘Severe TEAEs’ include those reported for ≥2% of all patients in the ‘All cycles’ column. COVID-19, coronavirus disease 2019; IgG, immunoglobulin G; MG, myasthenia gravis; RLZ, rozanolixizumab; TEAE, treatment-emergent adverse event; URTI, upper respiratory tract infection.

## Supplementary Table 6. MycarinG, MG0004 and MG0007 co-investigators and contributors

| Name | Location | Role | Contribution |
| --- | --- | --- | --- |
| Lela Tavzarashvili | Pineo Medical Ecosystem, Tbilisi, Georgia | Sub Investigator | Supported with the acquisition of data |
| Lotte Vinge | Aalborg Sygehus Nord, Denmark | Sub Investigator | Supported with the acquisition of data |
| Lotte Sahin Levison | Aarhus Universitetshospital, Denmark | Sub Investigator | Supported with the acquisition of data |
| Patimat Isabekova | Almazov National Medical Research Center, St. Petersburg, Russia | Sub Investigator | Supported with the acquisition of data |
| Matteo Garibaldi | AO S. Andrea, Università degli Studi di Roma La Sapienza, Italy | Sub Investigator | Supported with the acquisition of data |
| Antonio Lauletta | AO S. Andrea, Università degli Studi di Roma La Sapienza, Italy | Sub Investigator | Supported with the acquisition of data |
| Alessandra Parasole | AO S. Andrea, Università degli Studi di Roma La Sapienza, Italy | Study Coordinator | Supported with the acquisition of data |
| Rachana K. Gandhi Mehta | Atrium Health Wake Forest Baptist, Winston-Salem, NC, USA | Principal Investigator | Supported with the acquisition of data |
| Giovanni Antonini | Sapienza University of Rome, Department of Neurosciences, Mental Health and Sensory Organs (NESMOS), Italy | Principal Investigator | Supported with the acquisition of data |
| Elena Rossini | AOU Sant’Andrea Roma, Italy | Sub Investigator | Supported with the acquisition of data |
| Laura Tufano | AOU Sant’Andrea Roma, Italy | Sub Investigator | Supported with the acquisition of data |
| Luca Leonardi | AOU Sant’Andrea Roma, Italy | Sub Investigator | Supported with the acquisition of data |
| Laura Fionda | AOU Sant’Andrea Roma, Italy | Sub Investigator | Supported with the acquisition of data |
| Jean-Baptiste Chanson | Centre de Référence des Maladies Neuromusculaires NEIdF, Département de Neurologie, Hôpital de Hautepierre, Centre Hospitalier Universitaire de Strasbourg and ERO-NMD Strasbourg, Strasbourg, France | Sub Investigator | Supported with the acquisition of data |
| Leila Zaidi | Centre de Référence des Maladies Neuromusculaires NEIdF, Département de Neurologie, Hôpital de Hautepierre, Centre Hospitalier Universitaire de Strasbourg and ERO-NMD Strasbourg, Strasbourg, France | Study Coordinator | Supported with the acquisition of data |
| Genevieve Matte | Centre Hospitalier de l'Université de Montréal, Montreal, QC, Canada | Sub Investigator | Supported with the acquisition of data |
| Annie Dionne | CHU de Québec – Université Laval, Hôpital Enfant-Jésus, Québec, QC, Canada | Principal Investigator | Supported with the acquisition of data |
| Gordana Djordjevic | Clinic for Neurology, Clinical Centre Nis, Nis, Serbia | Principal Investigator | Supported with the acquisition of data |
| Aleksandar Stojanov | Clinic for Neurology, Clinical Centre Nis, Nis, Serbia | Sub Investigator | Supported with the acquisition of data |
| Francesco Habetswallner | Clinical Neurophysiology Unit, Cardarelli Hospital, Naples, Italy | Principal Investigator | Supported with the acquisition of data |
| Anna Piątek | Clinical Research Center Sp. z o.o., Medic-R Sp. K., Poland | Sub Investigator | Supported with the acquisition of data |
| Angela Genge | Clinical Research Unit, The Montreal Neurological Institute, Montreal, QC, Canada | Principal Investigator | Supported with the acquisition of data |
| Rami Massie | Clinical Research Unit, The Montreal Neurological Institute, Montreal, QC, Canada | Sub Investigator | Supported with the acquisition of data |
| Sonja Holm-Yildiz | Copenhagen Neuromuscular Center, Department of Neurology, Rigshospitalet, University of Copenhagen, Copenhagen, Denmark | Sub Investigator | Supported with the acquisition of data |
| Mads Stemmerik | Copenhagen Neuromuscular Center, Department of Neurology, Rigshospitalet, University of Copenhagen, Copenhagen, Denmark | Sub Investigator | Supported with the acquisition of data |
| Nanna Witting | Copenhagen Neuromuscular Center, Department of Neurology, Rigshospitalet, University of Copenhagen, Copenhagen, Denmark | Sub Investigator | Supported with the acquisition of data |
| Radwa Aly | Department of Neurology & Rehabilitation Medicine, George Washington University, Washington, DC, USA | Study Coordinator | Supported with the acquisition of data |
| Michaela Týblová | Department of Neurology and Center of Clinical Neuroscience First Faculty of Medicine Charles University and General Hospital, Prague, Czech Republic | Principal Investigator | Supported with the acquisition of data |
| Izabella Obál | Department of Neurology, Aalborg University Hospital, Aalborg, Denmark | Principal Investigator | Supported with the acquisition of data |
| Henning Andersen | Department of Neurology, Aarhus University Hospital, Aarhus, Denmark | Principal Investigator | Supported with the acquisition of data |
| Brandy Quarles | Department of Neurology, Augusta University, Augusta, GA, USA | Study Coordinator | Supported with the acquisition of data |
| Michael H. Rivner | Department of Neurology, Augusta University, Augusta, GA, USA | Principal Investigator | Supported with the acquisition of data |
| Akiyuki Uzawa | Department of Neurology, Chiba University Hospital, Chiba, Japan | Principal Investigator | Supported with the acquisition of data |
| Hiroyuki Naito | Department of Neurology, Hiroshima City Hiroshima Citizens Hospital, Hiroshima, Japan | Principal Investigator | Supported with the acquisition of data |
| Takamichi Sugimoto | Department of Neurology, Hiroshima City Hiroshima Citizens Hospital, Hiroshima, Japan | Principal Investigator | Supported with the acquisition of data |
| Kazumasa Yokoyama | Department of Neurology, Juntendo University Hospital, Tokyo, Japan | Principal Investigator | Supported with the acquisition of data |
| Konrad Rejdak | Department of Neurology, Medical University of Lublin, Lublin, Poland | Principal Investigator | Supported with the acquisition of data |
| Sebastian Szklener | Department of Neurology, Medical University of Lublin, Lublin, Poland | Sub Investigator | Supported with the acquisition of data |
| Dariusz Baranowski | Department of Neurology, Medical University of Lublin, Lublin, Poland | Sub Investigator | Supported with the acquisition of data |
| Jacek Kaźmierski | Clinical Research Center Sp. z o.o., Medic-R Sp. K., Poland | Study Coordinator | Supported with the acquisition of data |
| Claudia Schwering | Department of Neurology, Münster University Hospital, Germany | Sub Investigator | Supported with the acquisition of data |
| Christopher Nelke | Department of Neurology, Münster University Hospital, Germany | Sub Investigator | Supported with the acquisition of data |
| Marc Pawlitzki | Department of Neurology, Münster University Hospital, Germany | Sub Investigator | Supported with the acquisition of data |
| Yuri Fukushige | Department of Neurology, Nagasaki University Hospital, Nagasaki, Japan | Study Coordinator | Supported with the acquisition of data |
| Seiko Yoshida | Department of Neurology, Nagasaki University Hospital, Nagasaki, Japan | Study Coordinator | Supported with the acquisition of data |
| Shunsuke Yoshimura | Department of Neurology, Nagasaki University Hospital, Nagasaki, Japan | Principal Investigator | Supported with the acquisition of data |
| Eiko Uenaka | Department of Neurology, Osaka University Hospital, Osaka, Japan | Study Coordinator | Supported with the acquisition of data |
| Amy Chen | Department of Neurology, University of South Florida Morsani College of Medicine, Tampa, FL, USA | Sub Investigator | Supported with the acquisition of data |
| Jana Junkerova | Fakultni nemocnice Ostrava – Neurologicka klinika, Ostrava, Czech Republic | Principal Investigator | Supported with the acquisition of data |
| Lorenzo Maggi | Fondazione Istituto di Ricovero e Cura a Carattere Scientifico, Istituto Neurologico Carlo Besta, Italy | Sub Investigator | Supported with the acquisition of data |
| Renato Mantegazza | Fondazione Istituto di Ricovero e Cura a Carattere Scientifico, Istituto Neurologico Carlo Besta, Italy | Principal Investigator | Supported with the acquisition of data |
| Rita Frangiamore | Fondazione Istituto di Ricovero e Cura a Carattere Scientifico, Istituto Neurologico Carlo Besta, Italy | Sub Investigator | Supported with the acquisition of data |
| Fiammetta Vanoli | Fondazione Istituto di Ricovero e Cura a Carattere Scientifico, Istituto Neurologico Carlo Besta, Italy | Sub Investigator | Supported with the acquisition of data |
| Carlo Antozzi | Fondazione Istituto di Ricovero e Cura a Carattere Scientifico, Istituto Neurologico Carlo Besta, Italy | Sub Investigator | Supported with the acquisition of data |
| Silvia Bonanno | Fondazione Istituto di Ricovero e Cura a Carattere Scientifico, Istituto Neurologico Carlo Besta, Italy | Sub Investigator | Supported with the acquisition of data |
| Annamaria Gallone | Fondazione Istituto di Ricovero e Cura a Carattere Scientifico, Istituto Neurologico Carlo Besta, Italy | Sub Investigator | Supported with the acquisition of data |
| Elena Rinaldi | Fondazione Istituto di Ricovero e Cura a Carattere Scientifico, Istituto Neurologico Carlo Besta, Italy | Study Coordinator | Supported with the acquisition of data |
| Pietro Businaro | Fondazione Mondino Istituto Neurologico Nazionale a Carattere Scientifico – IRCCS, Pavia, Italy | Sub Investigator | Supported with the acquisition of data |
| Matteo Gastaldi | Fondazione Mondino Istituto Neurologico Nazionale a Carattere Scientifico – IRCCS, Pavia, Italy | Principal Investigator | Supported with the acquisition of data |
| Federico Mazzacane | Fondazione Mondino Istituto Neurologico Nazionale a Carattere Scientifico – IRCCS, Pavia, Italy | Sub Investigator | Supported with the acquisition of data |
| Raffaele Iorio | Fondazione Policlinico Universitario Agostino Gemelli IRCCS, Rome, Italy | Principal Investigator | Supported with the acquisition of data |
| Kore Liow | Hawaii Pacific Neuroscience, Honolulu, HI, USA | Principal Investigator | Supported with the acquisition of data |
| Karima Ghorab | Hôpital Dupuytren, Centre Hospitalier Universitaire de Limoges, Limoges, France | Principal Investigator | Supported with the acquisition of data |
| Laurent Magy | Hôpital Dupuytren, Centre Hospitalier Universitaire de Limoges, Limoges, France | Sub Investigator | Supported with the acquisition of data |
| Elena Cortés Vicente | Hospital de la Santa Creu i Sant Pau, Barcelona, Spain | Principal Investigator | Supported with the acquisition of data |
| Luis Querol Gutiérrez | Hospital de la Santa Creu i Sant Pau, Barcelona, Spain | Sub Investigator | Supported with the acquisition of data |
| Nuria Vidal-Fernández | Hospital de la Santa Creu i Sant Pau, Barcelona, Spain | Study Coordinator | Supported with the acquisition of data |
| Carlos Casasnovas Pons | Hospital Universitari de Bellvitge-IDIBELL and CIBERER, Barcelona, Spain | Principal Investigator | Supported with the acquisition of data |
| Velina Nedkova-Hristova | Hospital Universitari de Bellvitge-IDIBELL and CIBERER, Barcelona, Spain | Sub Investigator | Supported with the acquisition of data |
| Valentina Velez-Santamaria | Hospital Universitari de Bellvitge-IDIBELL and CIBERER, Barcelona, Spain | Sub Investigator | Supported with the acquisition of data |
| Daniel Sánchez-Tejerina | Hospital Universitari Vall d’Hebron, Spain | Sub Investigator | Supported with the acquisition of data |
| Francisco José Navacerrada Barrero | Hospital Universitario Infanta Sofía, Spain | Sub Investigator | Supported with the acquisition of data |
| Sheetal Shroff | Houston Methodist Neurological Institute, Houston, TX, USA | Principal Investigator | Supported with the acquisition of data |
| Bing Liao | Houston Methodist Neurological Institute, Houston, TX, USA | Sub Investigator | Supported with the acquisition of data |
| Ericka Greene | Houston Methodist Neurological Institute, Houston, TX, USA | Principal Investigator | Supported with the acquisition of data |
| Ashley Anderson | Houston Methodist Neurological Institute, Houston, TX, USA | Sub Investigator | Supported with the acquisition of data |
| Cynthia Bodkin | Indiana University Health Neuroscience Center, Indianapolis, IN, USA | Sub Investigator | Supported with the acquisition of data |
| Angela Micheels | Indiana University Health Neuroscience Center, Indianapolis, IN, USA | Study Coordinator | Supported with the acquisition of data |
| Rita Rinaldi | IRCCS Istituto delle Scienze Neurologiche di Bologna, Bologna, Italy | Principal Investigator | Supported with the acquisition of data |
| Roberto D'Angelo | IRCCS Istituto delle Scienze Neurologiche di Bologna, Bologna, Italy | Sub Investigator | Supported with the acquisition of data |
| Ia Rukhadze | Israeli-Georgian Medical Research Clinic Helsicore, Tbilisi, Georgia | Principal Investigator | Supported with the acquisition of data |
| Christine Bindler | Klinik für Neurologie, Klinikum Oberberg, Gummersbach, Germany | Sub Investigator | Supported with the acquisition of data |
| Franz Blaes | Klinik für Neurologie, Klinikum Oberberg, Gummersbach, Germany | Principal Investigator | Supported with the acquisition of data |
| Vasilios Tsoutsikas | Klinik für Neurologie, Klinikum Oberberg, Gummersbach, Germany | Sub Investigator | Supported with the acquisition of data |
| Jan D. Lünemann | Klinik für Neurologie, Universitätsklinikum Münster, Münster, Germany | Sub Investigator | Supported with the acquisition of data |
| Stefanie Glaubitz | Klinik für Neurologie, Universitätsmedizin Göttingen, Göttingen, Germany | Sub Investigator | Supported with the acquisition of data |
| Stefanie Meyer | Klinik für Neurologie, Universitätsmedizin Göttingen, Göttingen, Germany | Sub Investigator | Supported with the acquisition of data |
| Margret Schwarz | Klinik für Neurologie, Universitätsmedizin Göttingen, Göttingen, Germany | Study Coordinator | Supported with the acquisition of data |
| Rachel Zeng | Klinik für Neurologie, Universitätsmedizin Göttingen, Göttingen, Germany | Sub Investigator | Supported with the acquisition of data |
| Jana Zschüntzsch | Klinik für Neurologie, Universitätsmedizin Göttingen, Göttingen, Germany | Principal Investigator | Supported with the acquisition of data |
| Christian Geis | Klinik für Neurologie, Universtitätsklinikum Jena, Jena, Germany | Principal Investigator | Supported with the acquisition of data |
| Annekathrin Roediger | Klinik für Neurologie, Universtitätsklinikum Jena, Jena, Germany | Sub Investigator | Supported with the acquisition of data |
| Robert Steinbach | Klinik für Neurologie, Universtitätsklinikum Jena, Jena, Germany | Sub Investigator | Supported with the acquisition of data |
| Florian Then Bergh | Klinik und Polklinik für Neurologie, University of Leipzig, Leipzig, Germany | Principal Investigator | Supported with the acquisition of data |
| Leonid Zaslavskiy | St. Petersburg Regional Clinical Hospital, Russia | Principal Investigator | Supported with the acquisition of data |
| Dali Kankava | LTD New Hospitals, Georgia | Sub Investigator | Supported with the acquisition of data |
| Khatuna Sitchinava | LTD New Hospitals, Georgia | Sub Investigator | Supported with the acquisition of data |
| Nana Kvirkvelia | LTD Petre Sarajishvili Institute of Neurology, Georgia | Sub Investigator | Supported with the acquisition of data |
| Elene Nebadze | LTD Petre Sarajishvili Institute of Neurology, Georgia | Sub Investigator | Supported with the acquisition of data |
| Nazibrola Botchorishvili | LTD Simon Khechinashvili University Hospital, Georgia | Sub Investigator | Supported with the acquisition of data |
| Elie Naddaf | Mayo Clinic, Rochester, MN, USA | Principal Investigator | Supported with the acquisition of data |
| Isela Hernandez | MDA ALS and Neuromuscular Center, University of California, Irvine, CA, USA | Study Coordinator | Supported with the acquisition of data |
| Tahseen Mozaffar | MDA ALS and Neuromuscular Center, University of California, Irvine, CA, USA | Sub Investigator | Supported with the acquisition of data |
| Stefan Gingele | Medizinische Hochschule Hannover, Germany | Sub Investigator | Supported with the acquisition of data |
| Nora Möhn | Medizinische Hochschule Hannover, Germany | Sub Investigator | Supported with the acquisition of data |
| Thomas Skripuletz | Medizinische Hochschule Hannover, Germany | Sub Investigator | Supported with the acquisition of data |
| Tomasz Berkowicz | Miejskie Centrum Medyczne JONSCHER im. dr Karola Jonschera w Łodzi, Lodz, Poland | Principal Investigator | Supported with the acquisition of data |
| Paulina Budzińska | Miejskie Centrum Medyczne JONSCHER im. dr Karola Jonschera w Łodzi, Lodz, Poland | Sub Investigator | Supported with the acquisition of data |
| Francisca Iniesta | Multiple Sclerosis and Clinical Neuroimmunology Unit, Neurology Department, Hospital Clínico Universitario Virgen de la Arrixaca, Murcia, Spain | Study Coordinator | Supported with the acquisition of data |
| Gabriel Valero López | Multiple Sclerosis and Clinical Neuroimmunology Unit, Neurology Department, Hospital Clínico Universitario Virgen de la Arrixaca, Murcia, Spain | Sub Investigator | Supported with the acquisition of data |
| José Meca Lallana | Multiple Sclerosis and Clinical Neuroimmunology Unit, Neurology Department, Hospital Clínico Universitario Virgen de la Arrixaca, Murcia, Spain | Principal Investigator | Supported with the acquisition of data |
| Ivo Bozovic | Neurology Clinic, University Clinical Center of Serbia, Faculty of Medicine, University of Belgrade, Belgrade, Serbia | Sub Investigator | Supported with the acquisition of data |
| Dragana Lavrnic | Neurology Clinic, University Clinical Center of Serbia, Faculty of Medicine, University of Belgrade, Belgrade, Serbia | Sub Investigator | Supported with the acquisition of data |
| Aleksa Palibrk | Neurology Clinic, University Clinical Center of Serbia, Faculty of Medicine, University of Belgrade, Belgrade, Serbia | Sub Investigator | Supported with the acquisition of data |
| Stojan Peric | Neurology Clinic, University Clinical Center of Serbia, Faculty of Medicine, University of Belgrade, Belgrade, Serbia | Principal Investigator | Supported with the acquisition of data |
| Sophie Demeret | Neurology Department, Pitié Salpêtrière-Charles Foix Hospital Group, AP-HP, Sorbonne University, Paris, France | Principal Investigator | Supported with the acquisition of data |
| Loïc Le Guennec | Neurology Department, Pitié Salpêtrière-Charles Foix Hospital Group, AP-HP, Sorbonne University, Paris, France | Sub Investigator | Supported with the acquisition of data |
| Giorgia Querin | Neurology Department, Pitié Salpêtrière-Charles Foix Hospital Group, AP-HP, Sorbonne University, Paris, France | Sub Investigator | Supported with the acquisition of data |
| Tanya Stojkovic | Neurology Department, Pitié Salpêtrière-Charles Foix Hospital Group, AP-HP, Sorbonne University, Paris, France | Sub Investigator | Supported with the acquisition of data |
| Nicolas Weiss | Neurology Department, Pitié Salpêtrière-Charles Foix Hospital Group, AP-HP, Sorbonne University, Paris, France | Sub Investigator | Supported with the acquisition of data |
| Guilhem Solé | Neuromuscular Reference Center AOC, Nerve-Muscle Unit, Bordeaux University Hospitals, Bordeaux, France | Principal Investigator | Supported with the acquisition of data |
| Laurie Belin | Neuromuscular Reference Center AOC, Nerve-Muscle Unit, Bordeaux University Hospitals, Bordeaux, France | Study Coordinator | Supported with the acquisition of data |
| Marie-Hélène Violleau | Neuromuscular Reference Center AOC, Nerve-Muscle Unit, Bordeaux University Hospitals, Bordeaux, France | Study Coordinator | Supported with the acquisition of data |
| Temur Margania | New Hospitals, Tbilisi, Georgia | Principal Investigator | Supported with the acquisition of data |
| Vitalii Goldobin | North-Western State Medical University named after I.I. Mechnikov, St. Petersburg, Russia | Principal Investigator | Supported with the acquisition of data |
| Mustapha Itani | Odense University Hospital, Denmark | Sub Investigator | Supported with the acquisition of data |
| Tamar Vashadze | Pineo Medical Ecosystem, Tbilisi, Georgia | Sub Investigator | Supported with the acquisition of data |
| Alexander Tsiskaridze | Pineo Medical Ecosystem, Tbilisi, Georgia | Principal Investigator | Supported with the acquisition of data |
| Lucia Campetella | PU A. Gemelli, Università Cattolica del Sacro Cuore, Italy | Sub Investigator | Supported with the acquisition of data |
| Claudia Papi | PU A. Gemelli, Università Cattolica del Sacro Cuore, Italy | Sub Investigator | Supported with the acquisition of data |
| Gianvito Masi | Fondazione Policlinico Universitario Agostino Gemelli IRCCS, Rome, Italy | Sub Investigator | Supported with the acquisition of data |
| Nicolai Rasmus Preisler | Rigshospitalet, Denmark | Sub Investigator | Supported with the acquisition of data |
| Kandyda Derkacz - Kapala | Samodzielny Publiczny Szpital Kliniczny nr 4 w Lublinie, Poland | Sub Investigator | Supported with the acquisition of data |
| Eugenia Martínez-Hernández | Servicio de Neurología ICN, Hospital Clinic de Barcelona, Universitat de Barcelona, Barcelona, Spain | Principal Investigator | Supported with the acquisition of data |
| Montserrat Artola | Servicio de Neurología ICN, Hospital Clinic de Barcelona, Universitat de Barcelona, Barcelona, Spain | Study Coordinator | Supported with the acquisition of data |
| Yolanda Blanco Morgado | Servicio de Neurología ICN, Hospital Clinic de Barcelona, Universitat de Barcelona, Barcelona, Spain | Sub Investigator | Supported with the acquisition of data |
| Albert Saiz | Servicio de Neurología ICN, Hospital Clinic de Barcelona, Universitat de Barcelona, Barcelona, Spain | Principal Investigator | Supported with the acquisition of data |
| María Sepúlveda | Servicio de Neurología ICN, Hospital Clinic de Barcelona, Universitat de Barcelona, Barcelona, Spain | Sub Investigator | Supported with the acquisition of data |
| Gerardo Gutiérrez Gutiérrez | Neurology deparment, Hospital Universitario Infanta Sofía. Universidad Europea de Madrid. Department of Medicine, Faculty of Biomedical and Health Sciences, Madrid, Spain | Principal Investigator | Supported with the acquisition of data |
| Jiann-Horng Yeh | Department of Neurology, Shin Kong Wu Ho-Su Memorial Hospital, Taipei, Taiwan | Principal Investigator | Supported with the acquisition of data |
| Marina Janelidze | Simon Khechinashvili University Hospital, Tbilisi, Georgia | Principal Investigator | Supported with the acquisition of data |
| Denis Korobko | State Novosibirsk Regional Clinical Hospital, Novosibirsk, Russia | Principal Investigator | Supported with the acquisition of data |
| Kuan-Lin Lai | Taipei Veterans General Hospital, Taipei, Taiwan | Sub Investigator | Supported with the acquisition of data |
| Yi-Chung Lee | Taipei Veterans General Hospital, Taipei, Taiwan | Principal Investigator | Supported with the acquisition of data |
| Manuela Gambella | Université Côte d’Azur, Peripheral Nervous System and Muscle Department, Pasteur 2 Hospital, Centre Hospitalier Universitaire de Nice, Nice, France | Study Coordinator | Supported with the acquisition of data |
| Lubna Daniyal | University Health Network, Toronto, ON, Canada | Study Coordinator | Supported with the acquisition of data |
| Derrick Blackmore | University of Alberta, Edmonton, AB, Canada | Study Coordinator | Supported with the acquisition of data |
| Faraz S. Hussain | University of Alberta, Edmonton, AB, Canada | Study Coordinator | Supported with the acquisition of data |
| Zaeem A. Siddiqi | University of Alberta, Edmonton, AB, Canada | Principal Investigator | Supported with the acquisition of data |
| Hannah George | University of California, San Francisco Medical Center, San Francisco, CA, USA | Study Coordinator | Supported with the acquisition of data |
| Viktoriya Irodenko | University of California, San Francisco Medical Center, San Francisco, CA, USA | Sub Investigator | Supported with the acquisition of data |
| Min K. Kang | University of California, San Francisco Medical Center, San Francisco, CA, USA | Principal Investigator | Supported with the acquisition of data |
| Catherine Lomen-Hoerth | University of California, San Francisco Medical Center, San Francisco, CA, USA | Principal Investigator | Supported with the acquisition of data |
| Laura Rosow | University of California, San Francisco Medical Center, San Francisco, CA, USA | Sub Investigator | Supported with the acquisition of data |
| Kourosh Rezania | University of Chicago, Chicago, IL, USA | Principal Investigator | Supported with the acquisition of data |
| Zabeen Mahuwala | University of Kentucky, Lexington, KY, USA | Principal Investigator | Supported with the acquisition of data |
| Stephen Ryan | University of Kentucky, Lexington, KY, USA | Sub Investigator | Supported with the acquisition of data |
| Kai Su | University of Kentucky, Lexington, KY, USA | Study Coordinator | Supported with the acquisition of data |
| Renee Wagner | University of Kentucky, Lexington, KY, USA | Study Coordinator | Supported with the acquisition of data |
| Andrew Brown | University of Miami Miller School of Medicine, Miami, FL, USA | Sub Investigator | Supported with the acquisition of data |
| Khema R. Sharma | University of Miami Miller School of Medicine, Miami, FL, USA | Principal Investigator | Supported with the acquisition of data |
| Patricia Gonzalez Figueroa | University of Miami Miller School of Medicine, Miami, FL, USA | Study Coordinator | Supported with the acquisition of data |
| Chafic Karam | University of Pennsylvania, Philadelphia, PA, USA | Principal Investigator | Supported with the acquisition of data |
| Kelsey Moulton | University of Pennsylvania, Philadelphia, PA, USA | Study Coordinator | Supported with the acquisition of data |
| Salma Akhter | University of Southern California Keck School of Medicine, Los Angeles, CA, USA | Study Coordinator | Supported with the acquisition of data |
| Said R. Beydoun | University of Southern California Keck School of Medicine, Los Angeles, CA, USA | Principal Investigator | Supported with the acquisition of data |
| Maria Salvadó | Vall d’Hebron University Hospital, Passeig de la Vall d’Hebron, Barcelona, Spain | Sub Investigator | Supported with the acquisition of data |
| Olga Sidorova | Vladimirsky Moscow Regional Research and Clinical Institute, Moscow, Russia | Sub Investigator | Supported with the acquisition of data |
| Michala Jakubíková | Vseobecna fakultni nemocnice v Praze – Neurologicka klinika, Centrum pro diagnostiku a lecbu myasthenia gravis, Prague, Czech Republic | Sub Investigator | Supported with the acquisition of data |

Institutional Review Boards and Independent Ethics Committees

| **MG0003 IRB/IEC** | **Number of study sites** | **Date(s) of IRB/IEC approval(s)** |
| --- | --- | --- |
| Advarra, 372 Hollandview Trail Suite 300, Aurora, Ontario, L4G 0A5, Canada | 14 | 15 May 2019  10 Jul 2019  19 Jul 2019  22 Aug 2019  25 Sep 2019  25 Sep 2019  10 Oct 2019  04 Nov 2019  11 Dec 2019  31 Jan 2020  05 Feb 2020  07 Feb 2020  14 Feb 2020  20 Aug 2020 |
| Biological Sciences Division, Institutional Review Board, 5841S Maryland Avenue, MC7132, I-625, Chicago, Illinois, 60637, USA | 1 | 26 Nov 2019 |
| Chiba University Hospital, 1-8-1 Inohana, Chuo-ku, Chiba-shi, Chiba, 260-8677, Japan | 1 | 23 Mar 2020 |
| Comitato Etico Agostino Gemelli, Università Cattolica del Sacro Cuore, Largo Agostino Gemelli 8, 00168 Roma, Italy | 1 | 28 Oct 2019 |
| Comitato Etico - AVEC, Via Albertoni 15, 40138 Bologna, Italy | 1 | 01 Oct 2020 |
| Comitato Etico Azienda Ospedaliera Cardarelli  Via Cardarelli 9, 80131 Napoli, Italy | 1 | 12 Jun 2020 |
| Comitato Etico dell’Università Sapienza, Via di Grottarossa, 1035-1039 c/o Azienda Ospedaliera Sant’Andrea, 00189 Roma, Italy | 1 | 19 Dec 2019 |
| Comitato Etico Fondazione IRCCS Istituto Neurológico Besta, Via Celoria 11, 20133 Milano, Italy | 1 | 01 Oct 2019 |
| Comitato Etico Pavia, Viale Golgi 19, 27100 Pavia, Italy | 1 | 31 Jan 2020 |
| CPP Sud-Méditerranée II, Hôpital Sainte Marguerite, Pavillon 9–1er étage, 270 Boulevard Sainte Marguerite, Marseille, 13274, France | 7 | 08 Nov 2019  08 Nov 2019  08 Nov 2019  08 Nov 2019  08 Nov 2019  10 Jan 2020  06 Nov 2020 |
| De Videnskabsetiske Komitéer for Region, Midtylland, Skottenborg 26, 8800 Viborg, Denmark | 4 | 06 Sep 2019  06 Sep 2019  06 Sep 2019  08 Oct 2020 |
| Ethics Committee of Serbia, Vojvode Stepe 458 Street, Belgrade, 11221, Serbia | 2 | 04 Sep 2020 |
| Ethisch Comité UZA, Wilrijkstraat 10, 2650, Edegem, Belgium | 1 | 14 Oct 2019 |
| Ethikkommission an der Medizinischen Fakultät der Universität Leipzig, Liebigstr 18, 04103 Leipzig, Germany (local EC)  Ethikkommission der Friederich-Schiller-Universität Jena, Bachstraße 18, 07740 Jena, Germany (central EC) | 1 | 15 Nov 2019 |
| Ethikkommission der Ärztekammer Nordrhein, Tersteegenstraße 9, 40474 Düsseldorf, Germany (local EC)  Ethikkommission der Friederich-Schiller-Universität Jena, Bachstraße 18, 07740 Jena, Germany (central EC) | 1 | 20 Jan 2020 |
| Ethikkommission der Ärztekammer Westfalen-Lippe und der medizinischen Fakultät der WWU Münster, Gartenstr. 210–214, 48147 Münster, Germany (local EC)  Ethikkommission der Friederich-Schiller-Universität Jena, Bachstraße 18, 07740 Jena, Germany (central EC) | 1 | 15 Nov 2019 |
| Ethikkommission der Friederich-Schiller-Universität Jena, Bachstraße 18, 07740 Jena, Germany | 1 | 15 Nov 2019 |
| Ethikkommission der Medizinischen Fakultät der Universität Duisburg-Essen, Universitätsklinikum Essen, Robert-Koch-Str. 9–11, 45147 Essen, Germany (local EC)  Ethikkommission der Friederich-Schiller-Universität Jena, Bachstraße 18, 07740 Jena, Germany (central EC) | 1 | 15 Nov 2019 |
| Ethikkommission der Universitätsmedizin Göttingen, Von-Siebold-Straße 3, 37075 Göttingen, Germany (local EC)  Ethikkommission der Friederich-Schiller-Universität Jena, Bachstraße 18, 07740 Jena, Germany (central EC) | 1 | 25 Feb 2020 |
| Eticka Komise Všeobecná fakultní nemocnice v Praze, Na Bojisti 1, 128 08 Praha 2, Czech Republic | 1 | 17 Oct 2019 |
| Fakultni Nemocnice Ostrava, 17. listopadu 1790, 708 52 Ostrava, Czech Republic | 1 | 17 Oct 2019 |
| Fundació de Gestió Sanitària Hospital de La Santa Creu i Sant Pau, Sant Antoni Claret 167, Barcelona, 08025, Spain | 6 | 24 Jul 2019  24 Jul 2019  24 Jul 2019  04 Dec 2019  04 Dec 2019  29 Jan 2020 |
| General Hanamaki Hospital, 4-56, Otayacho, Hanamaki-shi, Iwate, 025-0082, Japan | 1 | 25 Dec 2019 |
| Hiroshima City Hiroshima Citizens Hospital  7-33, Motomachi, Naka-ku, Hiroshima, Hiroshima, 730-8518, Japan | 1 | 29 Jan 2020 |
| Houston Methodist Research Institutional Review Board, 6670 Bertner Avenue, Houston, Texas, 77030, USA | 1 | 24 Sep 2019 |
| Institutional Review Board of Shin Kong Wu Ho-Su Memorial Hospital, 95 Wen-Chan Road, Shih-Lin District, Taipei, 111, Taiwan | 1 | 13 Feb 2020 |
| Institutional Review Board of Taipei Veterans General Hospital, 201 Shih-Pai Road, Sec. 2, Taipei, 11217, Taiwan | 1 | 19 Feb 2020 |
| Juntendo University Hospital, 3-1-3 Hongo, Bunkyo-ku, Tokyo, 113-8431, Japan | 1 | 28 Feb 2020 |
| Keio University Hospital, 35 Shinano-Machi, Shinjuku-ku, Tokyo, 160-8582, Japan | 1 | 12 May 2020 |
| Kobe University Hospital, 7-5-2 Kusunoki-cho,  Chuo-ku, Kobe, Hyogo, 650-0017, Japan | 1 | 17 Apr 2020 |
| Komisja Bioetyczna przy UM, Al. Raclawickie 1, 059 Lublin, Poland | 4 | 19 Dec 2019  19 Dec 2019  19 Dec 2019  19 Feb 2020 |
| Local Ethics Committee of LTD Israeli-Georgian Medical Research Clinic Helsicore, 13 Tevdore Mgvdeli Street, Tbilisi, 0112, Georgia | 1 | 05 Jun 2020 |
| Local Ethics Committee of LTD New Hospitals, 12 Krtsanisi Street/71 Gorgasil Street, Tbilisi, 0114, Georgia | 1 | 05 Jun 2020 |
| Local Ethics Committee of LTD Petre Sarajishvili Institute of Neurology, 13 Tevdore Mgvdeli Street, Tbilisi, 0122, Georgia | 1 | 05 Jun 2020 |
| Local Ethics Committee of LTD Pineo Medical Ecosystem, 93 Gorgasali Street, Tbilisi, 0114, Georgia | 1 | 05 Jun 2020 |
| Local Ethics Committee of LTD Simon Khechinashvili University Hospital, 29-31-33 Chavchavadze Avenue, Tbilisi, 0179, Georgia | 1 | 05 Jun 2020 |
| Mayo Clinic, 201 Building, Room 4-60, 200 First St., SW Rochester, Minnesota, 55905, USA | 1 | 20 Nov 2019 |
| McGill University Health Center Research Board,  3801 University Street, Room 686, Montreal, Quebec, H3A 2B4, Canada | 3 | 18 Mar 2020 |
| Medical Research Council Ethics Committee for Clinical Pharmacology, Alkotmány u. 25, Budapest, 1054, Hungary | 2 | 25 Sep 2019 |
| M.F. Vladimirsky Moscow Region, Ulitsa Shchepkina, 61/2, Moscow, 129110, Russia | 1 | 07 Nov 2019 |
| Nagasaki University Hospital, 1-7-1 Sakamoto, Nagasaki-shi, Nagasaki, 852-8501, Japan | 1 | 26 Feb 2020 |
| National Hospital Organization Sendai Medical Center, 2-11-12 Miyagino, Miyagino-ku, Sendai, Miyagi, 983-8520, Japan | 1 | 26 Mar 2020 |
| North-Western State Medical University, 41 Kirochnaya Ulitsa, Saint-Petersburg, 191015, Russia | 1 | 30 Oct 2019 |
| Oguz Novosibirsk State Region, 130 Nemirovicha-Danchenko Street, Novosibirsk, Novosibirsk Oblast, 630087, Russia | 1 | 16 Jul 2020 |
| Osaka University Hospital, 2-15 Yamadaoka Suita, Osaka, 565-0871, Japan | 1 | 25 Feb 2020 |
| Research Ethics Committee China Medical University & Hospital, 2 Yude Road, Taichung, 40447, Taiwan | 1 | 21 Jan 2020 |
| State Budgetary Institution, 2 Akkuratova Street, Saint-Petersburg, 197341, Russia | 1 | 17 Feb 2020 |
| State Budgetary Institution, 45 Prospekt Lunacharskogo, Saint-Petersburg, 194291, Russia | 1 | 21 Feb 2020 |
| Tokyo Medical University Hospital, 6-7-1, Nishi-Shinjuku, Shinjuku-ku, Tokyo, 160-0023, Japan | 1 | 12 May 2020 |
| University Health Network Research Ethics Board, 700 University Avenue, Hydro Building, 10/F Room 10-56, Toronto, Ontario, M5G 1Z5, Canada | 1 | 17 Jan 2020 |
| University of Alberta Health Research Ethics Board, 11313-98 Avenue NW, North Power Plant – West Entrance, Suite #2-01, University of Alberta North Campus, Edmonton, Alberta, T6G 2N2, Canada | 1 | 10 Nov 2020 |
| University of California Institutional Review Board, 1640 Marengo Street, Suite 700, Los Angeles, California, 90033, USA | 1 | 20 Aug 2019 |
| University Of Kentucky Medical Institutional Review Board, 315 Kinkead Hall, Lexington, Kentucky, 40506-0057, USA | 1 | 25 Sep 2019 |
| Wake Forest University Health Sciences, Medical Center Boulevard, Winston-Salem, North Carolina, 27157, USA | 1 | 28 Oct 2019 |
| Western Institutional Review Board, 1019 39th Avenue S.E. Suite 120, Puyallup, Washington, 98374, USA | 3 | 01 Aug 2019  03 Sep 2019  01 May 2020 |
| West Midlands - Edgbaston Research Ethics Committee, 3rd Floor Barlow House, Minshull Street, Manchester, M1 3DZ, United Kingdom | 1 | 14 Nov 2019 |
| **MG0004 IRB/IEC** | **Number of study sites** | **Date(s) of IRB/IEC approval(s)** |
| Advarra, 372 Hollandview Trail Suite 300, Aurora, Ontario, L4G 0A5, Canada | 7 | 26 Sep 2019  30 Sep 2019  28 Oct 2019  16 Jan 2020  31 Mar 2020  10 Apr 2020  10 Dec 2020 |
| Comitato Etico dell’Università Sapienza, Via di Grottarossa, 1035-1039 c/o Azienda Ospedaliera Sant’Andrea, 00189 Roma, Italy | 1 | 07 May 2020 |
| Comitato Etico Fondazione IRCCS Istituto Neurológico Besta, Via Celoria 11, 20133 Milano, Italy | 1 | 19 Nov 2019 |
| Comitato Etico Pavia, Viale Golgi 19, 27100 Pavia, Italy | 1 | 08 Apr 2020 |
| CPP Sud-Méditerranée II, Hôpital Sainte Marguerite, Pavillon 9–1er étage, 270 Boulevard Sainte Marguerite, Marseille, 13274, France | 4 | 27 Nov 2019 |
| De Videnskabsetiske Komitéer for Region, Midtylland, Skottenborg 26, 8800 Viborg, Denmark | 1 | 12 Dec 2019 |
| Ethikkommission an der Medizinischen Fakultät der Universität Leipzig, Liebigstr 18, 04103 Leipzig, Germany (local EC)  Ethikkommission der Friederich-Schiller-Universität Jena, Bachstraße 18, 07740 Jena, Germany (central EC) | 1 | 20 Apr 2020 |
| Ethikkommission der Ärztekammer Westfalen-Lippe und der medizinischen Fakultät der WWU Münster, Gartenstr. 210–214, 48147 Münster, Germany (local EC)  Ethikkommission der Friederich-Schiller-Universität Jena, Bachstraße 18, 07740 Jena, Germany (central EC) | 1 | 20 Apr 2020 |
| Ethikkommission der Friederich-Schiller-Universität Jena, Bachstraße 18, 07740 Jena, Germany | 1 | 20 Apr 2020 |
| Eticka Komise Všeobecná fakultní nemocnice v Praze, Na Bojisti 1, 128 08 Praha 2, Czech Republic (local EC)  Eticka Komise Fakultni Nemocnice Ostrava, 17. listopadu 1790, 708 52 Ostrava, Czech Republic (central EC) | 1 | 12 Dec 2019 |
| Ethikkommission der Universitätsmedizin Göttingen, Von-Siebold-Straße 3, 37075 Göttingen, Germany (local EC)  Ethikkommission der Friederich-Schiller-Universität Jena, Bachstraße 18, 07740 Jena, Germany (central EC) | 1 | 20 Apr 2020 |
| Fakultni Nemocnice Ostrava, 17. listopadu 1790, 708 52 Ostrava, Czech Republic | 1 | 28 Nov 2019 |
| Fundació de Gestió Sanitària Hospital de La Santa Creu i Sant Pau, Sant Antoni Claret 167, Barcelona, 08025, Spain | 2 | 13 Nov 2019 |
| General Hanamaki Hospital, 4-56, Otayacho, Hanamaki-shi, Iwate, 025-0082, Japan | 1 | 18 Mar 2020 |
| Hiroshima City Hiroshima Citizens Hospital  7-33, Motomachi, Naka-ku, Hiroshima, Hiroshima, 730-8518, Japan | 1 | 25 Mar 2020 |
| Institutional Review Board of Shin Kong Wu Ho-Su Memorial Hospital, 95 Wen-Chan Road, Shih-Lin District, Taipei, 111, Taiwan | 1 | 09 Apr 2020 |
| Institutional Review Board of Taipei Veterans General Hospital, 201 Shih-Pai Road, Sec. 2, Taipei, 11217, Taiwan | 1 | 10 Apr 2020 |
| Keio University Hospital, 35 Shinano-Machi, Shinjuku-ku, Tokyo, 160-8582, Japan | 1 | 12 May 2020 |
| Komisja Bioetyczna przy UM, Al. Raclawickie 1, 059 Lublin, Poland | 3 | 27 Feb 2020 |
| McGill University Health Center Research Board,  3801 University Street, Room 686, Montreal, Quebec, H3A 2B4, Canada | 2 | 30 Jun 2020 |
| M.F. Vladimirsky Moscow Region, Ulitsa Shchepkina, 61/2, Moscow, 129110, Russia | 1 | 13 Feb 2020 |
| National Hospital Organization Sendai Medical Center, 2-11-12 Miyagino, Miyagino-ku, Sendai, Miyagi, 983-8520, Japan | 1 | 28 May 2020 |
| North-Western State Medical University, 41 Kirochnaya Ulitsa, Saint-Petersburg, 191015, Russia | 1 | 22 Jan 2020 |
| Osaka University Hospital, 2-15 Yamadaoka Suita, Osaka, 565-0871, Japan | 1 | 28 Apr 2020 |
| State Budgetary Institution, 2 Akkuratova Street, Saint-Petersburg, 197341, Russia | 1 | 17 Feb 2020 |
| State Budgetary Institution, 45 Prospekt Lunacharskogo, Saint-Petersburg, 194291, Russia | 1 | 21 Feb 2020 |
| University Health Network Research Ethics Board, 700 University Avenue, Hydro Building, 10/F Room 10-56, Toronto, Ontario, M5G 1Z5, Canada | 1 | 19 Jun 2020 |
| University of California Institutional Review Board, 1640 Marengo Street, Suite 700, Los Angeles, California, 90033, USA | 1 | 21 Nov 2019 |
| University Of Kentucky Medical Institutional Review Board, 315 Kinkead Hall, Lexington, Kentucky, 40506-0057, USA | 1 | 25 Sep 2019 |
| Wake Forest University Health Sciences, Medical Center Boulevard, Winston-Salem, North Carolina, 27157, USA | 1 | 15 Jan 2020 |
| Western Institutional Review Board, 1019 39th Avenue S.E. Suite 120, Puyallup, Washington, 98374, USA | 1 | 13 Nov 2019 |
| **MG0007 IRB/IEC** | **Number of study sites** | **Date(s) of IRB/IEC approval(s)** |
| Advarra, 372 Hollandview Trail Suite 300, Aurora, Ontario, L4G 0A5, Canada | 10 | 01 Dec 2020  01 Dec 2020  20 Jan 2021  01 Feb 2021  03 Mar 2021  14 Apr 2021  04 May 2021  04 May 2021  09 Jun 2021  29 Jun 2021 |
| Chiba University Hospital, 1-8-1 Inohana, Chuo-ku, Chiba-shi, Chiba, 260-8677, Japan | 1 | 21 Dec 2020 |
| Comitato Etico Agostino Gemelli, Università Cattolica del Sacro Cuore, Largo Agostino Gemelli 8, 00168 Roma, Italy | 1 | 26 Apr 2021 |
| Comitato Etico - AVEC, Via Albertoni 15, 40138 Bologna, Italy | 1 | 22 Jun 2021 |
| Comitato Etico Azienda Ospedaliera Cardarelli  Via Cardarelli 9, 80131 Napoli, Italy | 1 | 23 Feb 2021 |
| Comitato Etico dell’Università Sapienza, Via di Grottarossa, 1035-1039 c/o Azienda Ospedaliera Sant’Andrea, 00189 Roma, Italy | 1 | 30 Apr 2021 |
| Comitato Etico Fondazione IRCCS Istituto Neurológico Besta, Via Celoria 11, 20133 Milano, Italy | 1 | 01 Dec 2020 |
| Comitato Etico Pavia, Viale Golgi 19, 27100 Pavia, Italy | 1 | 15 Apr 2021 |
| CPP Sud-Méditerranée II, Hôpital Sainte Marguerite, Pavillon 9–1er étage, 270 Boulevard Sainte Marguerite, Marseille, 13274, France | 5 | 10 Feb 2021 |
| De Videnskabsetiske Komitéer for Region, Midtylland, Skottenborg 26, 8800 Viborg, Denmark | 3 | 14 Dec 2020 |
| Ethics Committee of Serbia, Vojvode Stepe 458 Street, Belgrade, 11221, Serbia | 1 | 28 Jan 2021 |
| Ethikkommission an der Medizinischen Fakultät der Universität Leipzig, Liebigstr 18, 04103 Leipzig, Germany (local EC)  Ethikkommission der Friederich-Schiller-Universität Jena, Bachstraße 18, 07740 Jena, Germany (central EC) | 1 | 02 Dec 2020 |
| Ethikkommission der Ärztekammer Westfalen-Lippe und der medizinischen Fakultät der WWU Münster, Gartenstr. 210–214, 48147 Münster, Germany (local EC)  Ethikkommission der Friederich-Schiller-Universität Jena, Bachstraße 18, 07740 Jena, Germany (central EC) | 1 | 02 Dec 2020 |
| Ethikkommission der Friederich-Schiller-Universität Jena, Bachstraße 18, 07740 Jena, Germany | 1 | 02 Dec 2020 |
| Ethikkommission der Medizinischen Fakultät der Universität Duisburg-Essen, Universitätsklinikum Essen, Robert-Koch-Str. 9–11, 45147 Essen, Germany (local EC)  Ethikkommission der Friederich-Schiller-Universität Jena, Bachstraße 18, 07740 Jena, Germany (central EC) | 1 | 15 Jun 2021 |
| Ethikkommission der Universitätsmedizin Göttingen, Von-Siebold-Straße 3, 37075 Göttingen, Germany (local EC)  Ethikkommission der Friederich-Schiller-Universität Jena, Bachstraße 18, 07740 Jena, Germany (central EC) | 1 | 02 Dec 2020 |
| Eticka Komise Všeobecná fakultní nemocnice v Praze, Na Bojisti 1, 128 08 Praha 2, Czech Republic | 1 | 15 Oct 2020 |
| Fakultni Nemocnice Ostrava, 17. listopadu 1790, 708 52 Ostrava, Czech Republic | 1 | 24 Sep 2020 |
| Fundació de Gestió Sanitària Hospital de La Santa Creu i Sant Pau, Sant Antoni Claret 167, Barcelona, 08025, Spain | 4 | 14 Oct 2020 |
| General Hanamaki Hospital, 4-56, Otayacho, Hanamaki-shi, Iwate, 025-0082, Japan | 1 | 16 Dec 2020 |
| Hiroshima City Hiroshima Citizens Hospital  7-33, Motomachi, Naka-ku, Hiroshima, Hiroshima, 730-8518, Japan | 1 | 27 Jan 2021 |
| Houston Methodist Research Institutional Review Board, 6670 Bertner Avenue, Houston, Texas, 77030, USA | 1 | 12 Oct 2021 |
| Institutional Review Board of Shin Kong Wu Ho-Su Memorial Hospital, 95 Wen-Chan Road, Shih-Lin District, Taipei, 111, Taiwan | 1 | 27 Nov 2020 |
| Institutional Review Board of Taipei Veterans General Hospital, 201 Shih-Pai Road, Sec. 2, Taipei, 11217, Taiwan | 1 | 10 Dec 2020 |
| Juntendo University Hospital, 3-1-3 Hongo, Bunkyo-ku, Tokyo, 113-8431, Japan | 1 | 15 Dec 2020 |
| Keio University Hospital, 35 Shinano-Machi, Shinjuku-ku, Tokyo, 160-8582, Japan | 1 | 04 Feb 2021 |
| Kobe University Hospital, 7-5-2 Kusunoki-cho,  Chuo-ku, Kobe, Hyogo, 650-0017, Japan | 1 | 15 Feb 2021 |
| Komisja Bioetyczna przy UM, Al. Raclawickie 1, 059 Lublin, Poland | 4 | 26 Nov 2020 |
| Local Ethics Committee of LTD Israeli-Georgian Medical Research Clinic Helsicore, 13 Tevdore Mgvdeli Street, Tbilisi, 0112, Georgia | 1 | 28 Oct 2020 |
| Local Ethics Committee of LTD New Hospitals, 12 Krtsanisi Street/71 Gorgasil Street, Tbilisi, 0114, Georgia | 1 | 29 Oct 2020 |
| Local Ethics Committee of LTD Petre Sarajishvili Institute of Neurology, 13 Tevdore Mgvdeli Street, Tbilisi, 0122, Georgia | 1 | 28 Oct 2020 |
| Local Ethics Committee of LTD Pineo Medical Ecosystem, 93 Gorgasali Street, Tbilisi, 0114, Georgia | 1 | 29 Oct 2020 |
| Local Ethics Committee of LTD Simon Khechinashvili University Hospital, 29-31-33 Chavchavadze Avenue, Tbilisi, 0179, Georgia | 1 | 28 Oct 2020 |
| McGill University Health Center Research Board,  3801 University Street, Room 686, Montreal, Quebec, H3A 2B4, Canada | 3 | 07 Jul 2021 |
| Nagasaki University Hospital, 1-7-1 Sakamoto, Nagasaki-shi, Nagasaki, 852-8501, Japan | 1 | 17 Feb 2021 |
| National Hospital Organization Sendai Medical Center, 2-11-12 Miyagino, Miyagino-ku, Sendai, Miyagi, 983-8520, Japan | 1 | 24 Dec 2020 |
| North-Western State Medical University, 41 Kirochnaya Ulitsa, Saint-Petersburg, 191015, Russia | 1 | 03 Feb 2021 |
| Oguz Novosibirsk State Region, 130 Nemirovicha-Danchenko Street, Novosibirsk, Novosibirsk Oblast, 630087, Russia | 1 | 09 Mar 2021 |
| Osaka University Hospital, 2-15 Yamadaoka Suita, Osaka, 565-0871, Japan | 1 | 16 Feb 2021 |
| Research Ethics Committee China Medical University & Hospital, 2 Yude Road, Taichung, 40447, Taiwan | 1 | 24 Nov 2020 |
| State Budgetary Institution, 2 Akkuratova Street, Saint-Petersburg, 197341, Russia | 1 | 15 Feb 2021 |
| State Budgetary Institution, 45 Prospekt Lunacharskogo, Saint-Petersburg, 194291, Russia | 1 | 12 Feb 2021 |
| Tokyo Medical University Hospital, 6-7-1, Nishi-Shinjuku, Shinjuku-ku, Tokyo, 160-0023, Japan | 1 | 15 Deb 2020 |
| University Health Network Research Ethics Board, 700 University Avenue, Hydro Building, 10/F Room 10-56, Toronto, Ontario, M5G 1Z5, Canada | 1 | 21 Jun 2021 |
| University of Alberta Health Research Ethics Board, 11313-98 Avenue NW, North Power Plant – West Entrance, Suite #2-01, University of Alberta North Campus, Edmonton, Alberta, T6G 2N2, Canada | 1 | 14 Sep 2021 |
| University Of Kentucky Medical Institutional Review Board, 315 Kinkead Hall, Lexington, Kentucky, 40506-0057, USA | 1 | 10 Mar 2021 |
| Wake Forest University Health Sciences, Medical Center Boulevard, Winston-Salem, North Carolina, 27157, USA | 1 | 10 Feb 2021 |
|  |  |  |

EC, Ethics Committee; IEC, Independent Ethics Committee; IRB, Institutional Review Board.
